# Lineage-specific control of TFIIH by MITF determines transcriptional homeostasis and DNA repair

**DOI:** 10.1038/s41388-018-0661-x

**Published:** 2019-01-16

**Authors:** Marcos Seoane, Sophia Buhs, Pablo Iglesias, Julia Strauss, Ann-Christin Puller, Jürgen Müller, Helwe Gerull, Susanne Feldhaus, Malik Alawi, Johanna M. Brandner, Dennis Eggert, Jinyan Du, Jürgen Thomale, Peter J. Wild, Martin Zimmermann, Thomas Sternsdorf, Udo Schumacher, Peter Nollau, David E. Fisher, Martin A. Horstmann

**Affiliations:** 1 Research Institute Children’s Cancer Center Hamburg, Hamburg, 20246 Germany; 2 Department of Pediatric Hematology and Oncology, University Medical Center Hamburg, Hamburg, 20246 Germany; 3 Institute of Anatomy and Experimental Morphology, University Medical Center Hamburg, Hamburg, 20246 Germany; 4 Bioinformatics Service Facility, University Medical Center Hamburg, Hamburg, 20246 Germany; 5 Heinrich Pette Institute, Leibniz Institute for Experimental Virology, Hamburg, 20251 Germany; 6 Department of Dermatology, University Medical Center Hamburg, Hamburg, 20246 Germany; 7 Max-Planck-Institute for the Structure and Dynamics of Matter, Hamburg, 22761 Germany; 8 Department of Pediatric Oncology, Dana Farber Cancer Institute, Harvard Medical School, Boston, MA 02115 USA; 9 Institute of Cell Biology, University Duisburg-Essen, Essen, 45122 Germany; 10 Institute of Surgical Pathology, University Hospital Zürich, Zürich, 8091 Switzerland; 11 Department of Pediatric Hematology and Oncology, Medical School Hannover, Hannover, 30625 Germany; 12 Department of Dermatology, Cutaneous Biology Research Center, Massachusetts General Hospital, Harvard Medical School, Boston, MA 02115 USA; 13 Present Address: Merrimack Pharmaceuticals, Cambridge, MA 02139 USA

**Keywords:** Melanoma, Mechanisms of disease, Nucleotide excision repair, Transcription

## Abstract

The melanocytic lineage, which is prominently exposed to ultraviolet radiation (UVR) and radiation-independent oxidative damage, requires specific DNA-damage response mechanisms to maintain genomic and transcriptional homeostasis. The coordinate lineage-specific regulation of intricately intertwined DNA repair and transcription is incompletely understood. Here we demonstrate that the Microphthalmia-associated transcription factor (MITF) directly controls general transcription and UVR-induced nucleotide excision repair by transactivation of *GTF2H1* as a core element of TFIIH. Thus, MITF ensures the rapid resumption of transcription after completion of strand repair and maintains transcriptional output, which is indispensable for survival of the melanocytic lineage including melanoma in vitro and in vivo. Moreover, MITF controls c-MYC implicated in general transcription by transactivation of far upstream binding protein 2 (FUBP2/KSHRP), which induces c-MYC pulse regulation through TFIIH, and experimental depletion of MITF results in consecutive loss of CDK7 in the TFIIH-CAK subcomplex. Targeted for proteasomal degradation, CDK7 is dependent on transactivation by MITF or c-MYC to maintain a steady state. The dependence of TFIIH-CAK on sequence-specific MITF and c-MYC constitutes a previously unrecognized mechanism feeding into super-enhancer-driven or other oncogenic transcriptional circuitries, which supports the concept of a transcription-directed therapeutic intervention in melanoma.

## Introduction

Survival, proliferation, and differentiation of the melanocytic lineage are determined by the Microphthalmia-associated transcription factor (MITF) [1–3]. MITF’s pivotal role in lineage survival is underscored by the observations that its mutation entails a substantial loss of melanocyte viability and its expression is largely maintained upon malignant transformation [4, 5]. MITF’s impact on lineage survival is partially explained by direct transcriptional control of the anti-apoptotic BCL-2 [1]. In the cell cycle, MITF exerts opposing context-dependent functions by direct regulation of CDK2 vs. INK4 and p21Cip1 [2, 6, 7]. In regard to differentiation, MITF is essential to UV-protective pigmentation which is induced by the p53-dependent Melanocyte Stimulating Hormone [3, 8, 9].

Mechanistically, it is still incompletely understood how MITF is linked to melanomagenesis. In 5–20% of human melanomas *MITF* undergoes genomic amplification and as such it acquires features of a lineage-survival oncogene [10]. In addition, a SUMOylation-defective MITF germline mutation MITF-E318K with increased transcriptional activity has been identified, which predisposes to familial and sporadic melanoma and renal cell carcinoma [11, 12]. MITF’s oncogenic role is further supported by its EWS-ATF1 dependent upregulation in clear cell sarcoma, which is indispensable for survival and growth of the sarcoma [13]. By contrast, a subset of bulk melanomas (<20%) reveal a low abundance of MITF, which has been linked to an invasive, treatment-resistant phenotype [14]. In addition, single-cell expression analyses identified melanoma cells with a low MITF/AXL ratio in MITF-high bulk melanomas, which may be able to evade senescence and confer treatment resistance [15, 16].

Opposing data on MITF’s role in UV-dependent DNA damage response pathways and genomic stability have been published and the mechanistic link between MITF and nucleotide excision repair (NER) has not been clearly defined [17, 18]. Here we show that MITF impinges upon the functional interface of transcription and nucleotide excision repair (NER) embodied by the general transcription factor IIH [19, 20]. TFIIH is a multi-protein complex that is composed of the helicases XPB and XPD, subunits GTF2H1 (p62), p52, p44, p34, p8 (TTDA) which form the core complex as well as the CDK-activating kinase (CAK) sub-complex that contains CDK7, CCNH, and the assembly factor MAT1 [20, 21]. XPD bridges the core complex and the CAK sub-complex [22]. TFIIH is not only involved in basal transcription but also in nucleotide excision repair, transactivation of nuclear receptors and in the cell cycle through CDK7 activity of the CAK complex [23, 24]. At mitosis CDK1/Cyclin B phosphorylates CDK7 at serine 164 resulting in transcription inhibition and CAK dissociates from TFIIH prompted by degradation of the bridging element XPD [25]. As a TFIIH core element GTF2H1 physically interacts with TFIIE of the transcriptional pre-initiation complex [26]. In NER, GTF2H1 contacts DNA damage recognition factors XPC-HR23B and the 3ʹ-endonuclease XPG [27, 28].

Our studies identify a previously unrecognized mechanism how a lineage-restricted sequence-specific transcription factor controls genomic and transcriptional homeostasis through transactivation of GTF2H1 as a key component of the TFIIH transcription/repair apparatus. Importantly, the MITF–GTF2H1 axis is preserved in MITF-abundant melanomas with potential implications for primary tumor progression and macro-metastatic disease. Moreover, MITF regulates TFIIH kinase (CDK7), which is lost upon depletion of MITF but rescued by its structural homolog c-MYC thus adopting MITF’s role in transcriptional homeostasis at the expense of a melanocyte-specific program. The dependence of TFIIH-CAK on the sequence-specific transcriptional master regulators MITF or MYC constitutes a vulnerability in melanoma.

## Results

### MITF determines transcriptional activity and is linked to GTF2H1

**Fig. 1:**
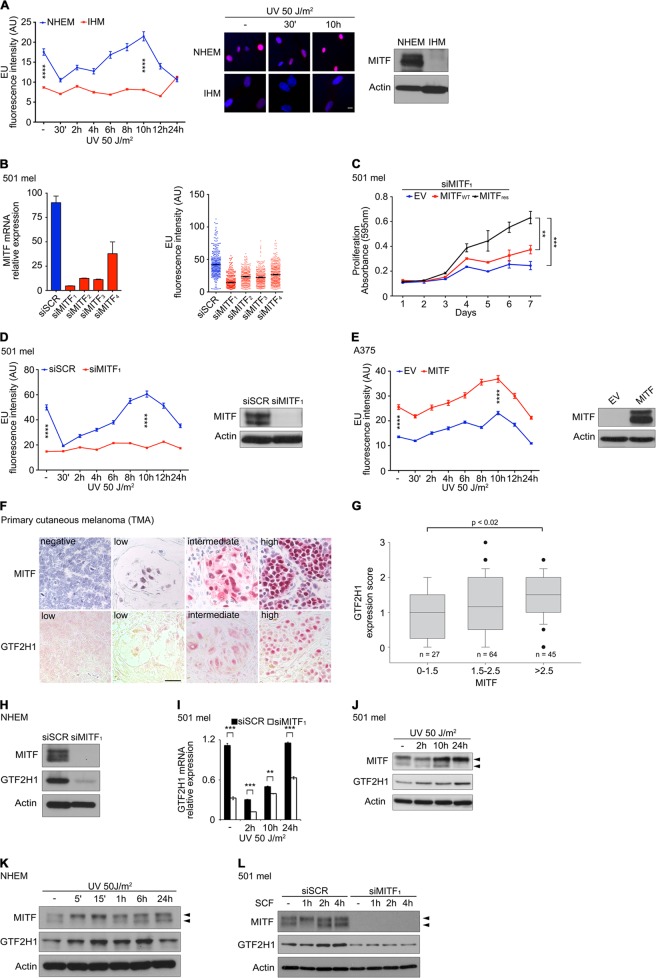
MITF regulates GTF2H1 and determines transcriptional output. **a** Left panel: time course of transcriptional activity detected by 5-ethynyl-uridine (EU) incorporation in neonatal human epidermal melanocytes (NHEM) vs. TERT-immortalized human melanocytes (IHM) after UVB irradiation. Middle panel: fluorescence microscopy depicting EU incorporation of single cells. Images were taken at indicated time points after UVR. Nuclear staining by Hoechst 33342 (blue), EU (red), scale: 10 µm. Right panel: immunoblot shows differential expression levels of MITF protein in NHEM and IHM. Actin used as loading control. **b** MITF mRNA expression (left) and EU incorporation (right) of 501 mel cells after transfection with four different MITF-directed siRNA vs. control siSCR. **c** Proliferation of 501 mel cells upon siMITF_1_ transfection and retroviral expression of wild-type MITF (MITF_WT_) and siRNA-resistant mutant MITF (MITF_RES_) vs. empty vector (EV). Graphs represent mean absorbance of crystal violet ± SD from technical triplicates (two-tailed unpaired *t*-test; **, *p* < 0.01; ***, *p* < 0.001). **d** EU incorporation of UVB irradiated 501 mel cells after siMITF_1_ vs. siSCR transfection. Immunoblot shows MITF protein levels after siRNA transfection. **e** EU incorporation of UVB irradiated A375 cells after retroviral expression of wild-type MITF or EV. Immunoblots reflect retrovirus-mediated MITF protein expression compared to EV in A375 cells. Graphs in **a**, **d**, and **e** indicate mean ±SEM of fluorescence intensity in ≥100 nuclei (****, *p* < 10^–4^, two-tailed unpaired *t*-test). **f** MITF and GTF2H1 protein expression in representative primary cutaneous melanomas (TMA, tissue microarray comprising *n* = 136 samples; scale: 50 µm). **g** Relationship between MITF and GTF2H1 protein expression in primary cutaneous melanomas (*p* = 0.0109 Pearson correlation coefficient). **h** Immunoblot analysis of MITF and GTF2H1 expression under siMITF_1_ vs. siSCR control in NHEM. **i** GTF2H1 mRNA expression under siMITF_1_ vs. control siRNA after UVB irradiation of 501 mel cells. Mean ± SD normalized to GAPDH is presented (**, *p* < 0.01; ***, *p* < 0.001; two-tailed unpaired *t*-test). **j** Time course of MITF and GTF2H1 protein expression after UVB irradiation of 501 mel cells showing an increase in phosphorylated MITF (marked by upper arrowhead; lower arrowhead, unphosphorylated MITF) and in GTF2H1. **k** Immunoblot of MITF and GTF2H1 expression after UVR of NHEM including early time points (5ʹ, 15’) displaying a rapid increase in phosphorylated MITF accompanied by an up-regulation of GTF2H1. **l** MITF and GTF2H1 protein expression after SCF stimulation in 501 mel cells transfected with siMITF_1_ vs. control siSCR. Note rapid and transient shift of MITF to slower migrating phosphorylated form. **j**–**l**: lower and upper arrowheads indicate non-phosphorylated MITF at 54 kd and phosphorylated MITF at 60 kd, respectively. Experiments depicted in **a**–**e**, **h**–**l** were performed at least twice with very similar results

To test the hypothesis whether MITF acts at the interface of DNA repair and transcription we first assessed the transcription rate of melanocytes upon UVB irradiation (UVR)-mediated genotoxic attack by incorporation of 5-ethynyl-uridine (EU) in the presence or (near) absence of MITF [29]. UVR treatment of MITF-positive neonatal human epidermal melanocytes (NHEM) led to a substantial but transient reduction in transcriptional activity generally due to stalled RNA polymerases at DNA lesions and subsequent transcription-coupled repair (TCR) [30]. By contrast, loss of constitutive expression of MITF in telomerase (TERT)-immortalized human melanocytes (IHM) was associated with low transcription rates notwithstanding a faster replication of IHM compared to NHEM (Fig. 1a). In addition, experimental depletion of MITF by RNA interference (RNAi) in 501 mel cells using multiple independent siRNA was associated with a strong reduction in basal transcription (Fig. 1b). On-target specificity of the most efficient MITF-directed siRNA (siMITF_1_) was validated by retroviral expression of an RNA-interference resistant MITF carrying triple silent mutations, which abolished the senescence-like phenotype of MITF depletion (Fig. 1c and Supplementary Figures 1a-c) [17]. In analogy to MITF-negative IHM, UVR treatment of MITF-depleted 501 mel cells did not elicit an oscillatory transcriptional activity in contrast to control siRNA treated cells (Fig. 1d). In a reciprocal experiment, forced expression of MITF in MITF-negative A375 melanoma cells augmented basal transcriptional activity and transcriptional recovery after UVR (Fig. 1e). To screen for potential MITF target genes coding for general transcription and nucleotide excision repair (NER) factors, we analyzed transcriptome data on a panel of primary and metastatic melanomas, melanoma in situ and melanocytes (GSE7553). Masked by a dominating strictly MITF-dependent gene expression signature composed of MLANA, CDK2, PMEL, and other established MITF target genes in neighborhood analyses, we identified a subset of differentially regulated general transcription/NER genes using a less stringent comparative marker selection analysis (Supplementary Figure 2a). Among these we selected the general transcription factor 2H1 (GTF2H1) for further analysis, since it reflects the dual action of TFIIH in transcription initiation and NER [20, 31]. The concordant expression of MITF and GTF2H1 transcripts was confirmed in several genetically heterogeneous melanoma lines by RT-PCR (Supplementary Figure 2b and Supplementary Table 1). Moreover, immunohistochemical analyses of a large panel of primary human cutaneous melanomas (*n* = 136) spotted onto tissue microarrays (TMA) revealed a moderate but significant correlation between MITF and GTF2H1 at the protein level (Fig. 1f, g, Supplementary Figure 2c and Supplementary Table 2). RNAi-mediated depletion of MITF in melanocytes from various donors and melanoma cells using several independent siRNAs and expression of an adenovirus-driven dominant-negative MITF mutant resulted in a substantial reduction in constitutively expressed GTF2H1 at mRNA and protein levels, in particular under conditions of serum starvation (Fig. 1h and Supplementary Figures 2d-g). Upon UVR MITF-depletion was associated with markedly decreased GTF2H1 mRNA expression levels over time (Fig. 1i). At the protein level both MITF and GTF2H1 activities showed a clear induction after UVR treatment of primary melanocytes and 501 mel cells (Fig. 1j, k). Stem cell factor (SCF) has previously been shown to be secreted by UV-irradiated keratinocytes and to activate MITF via c-KIT/MAP kinase signaling in melanocytes [32, 33]. To examine GTF2H1 regulation in this c-KIT dependent context, we stimulated 501 mel cells with SCF and observed a phosphorylation-dependent MITF mobility shift to the slower migrating, transcriptionally active form and an increase in GTF2H1 expression, respectively. Conversely, the observed SCF-triggered induction of GTF2H1 was completely abrogated upon depletion of MITF (Fig. 1l).

### GTF2H1 is a direct transcriptional target of MITF

**Fig. 2:**
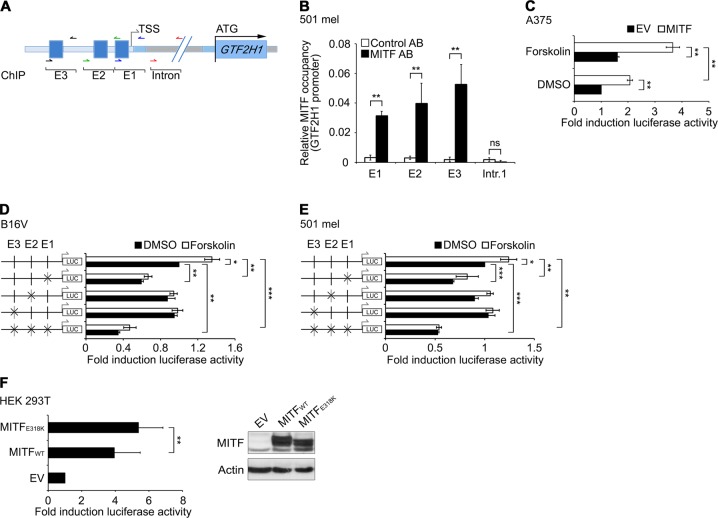
GTF2H1 is a direct transcriptional target of MITF. **a** Human *GTF2H1* locus with MITF-binding consensus sequences (E1–3) and ChIP primer annealing sites (colored arrows). TSS transcriptional start site, ATG, start codon. **b** ChIP, in vivo occupancy of MITF at GTF2H1 promoter in 501 mel cells. E box enrichment compared to intron 1. Mean ± SD from technical triplicates is presented. IgG (AB) was used as control antibody. **c** Normalized luciferase activity of GTF2H1 promoter after transfection with wild-type MITF vs. empty vector (EV) ± forskolin in A375 melanoma cells. Graphs represent mean ± SD from technical triplicates. **d**, **e** Normalized luciferase activity of GTF2H1 promoter containing E box site mutations in B16V or 501 mel cells ± forskolin. Mean ± SD from technical triplicates. **b**–**e** Experiments were performed twice with comparable results. **f** Transactivation of GTF2H1 promoter after wild-type MITF vs. SUMOylation-defective MITF-E318K transfection of HEK 293T cells as mean of fold-induction ± SD from 10 independent experiments (biological replicates). Right panel: immunoblot showing MITF protein levels in transfected HEK 293T cells. **b**–**f** *, *p* < 0.05; **, *p* < 0.01; ***, *p* < 0.001; two-tailed unpaired *t*-test)

To assess the directness of the underlying regulatory mechanism, we scrutinized the human GTF2H1 promoter for transcription factor binding sites. Beside SP1 and cAMP response element (CRE) sites, we identified three conserved elements that fit the E box consensus sequence (t)CAC/TGTG −34, −67, and −340 base pairs upstream of the transcriptional start site (TSS), which were occupied by MITF in ChIP assays (Fig. 2a, b). In cellular models devoid of MITF, the intact GTF2H1 promoter was clearly transactivated by exogenous MITF, exceeding CRE-binding protein (CREB)-mediated effects by treatment with the cAMP agonist forskolin (Fig. 2c). Individual site-specific mutation at the E boxes reduced GTF2H1 promoter activity and combined site-specific mutations at all three E boxes increased the repressive effect on reporter activity as demonstrated in murine and human melanoma cells (Fig. 2d, e). Notably, SUMOylation-defective MITF-E318K, previously implicated in predisposition to familial and sporadic melanoma, significantly enhanced GTF2H1 promoter activity compared to wild-type MITF (Fig. 2f) [11, 12].

### Regulation of GTF2H1 by MITF maintains transcriptional homeostasis

Beyond its essential role in TFIIH complex assembly and stability, the function of GTF2H1 with regard to transcriptional activity is poorly defined and it has not been linked to specific repair or transcription deficiency phenotypes in contrast to other TFIIH components such as XPB, XPD, and TTDA (GTF2H5) [34]. To independently confirm that differential regulation of GTF2H1 by MITF feeds back on global transcription, we performed RNA sequencing (RNA-seq) under siRNA-mediated knockdown of MITF or GTF2H1 vs. scrambled siRNA with spike-in normalization [35]. Transcriptomes were similarly composed and showed differential gene regulation upon normalization of relative expression levels, but revealed an overlapping almost universal repression upon depletion of MITF or GTF2H1 compared to controls as demonstrated by correction to cell equivalents (Supplementary Figure 3a). RNA-seq data were validated by qRT-PCR of randomly selected transcripts after normalizing mRNA input to cell number (Supplementary Figure 3b). In accordance with the established role of TFIIH in POLR2- and POLR1-dependent transcription, experimental disruption of the MITF–GTF2H1 axis resulted in a decrease in total, messenger and ribosomal RNA (Supplementary Figure 3c) [36, 37]. To minimize convolving cell cycle effects on global transcriptional output, we performed thymidine block experiments combined with RNA interference, which caused a significant expansion of non-cycling cell populations with low RNA content under MITF or GTF2H1 depletion compared to control siRNA_._ This finding indicates that MITF and its transcriptional target GTF2H1 have a profound impact on global transcriptional activity, which clearly exceeds the transcriptional demand of cell cycle activity (Supplementary Figure 3d).

### MITF enhances nucleotide excision repair through regulation of GTF2H1

**Fig. 3:**
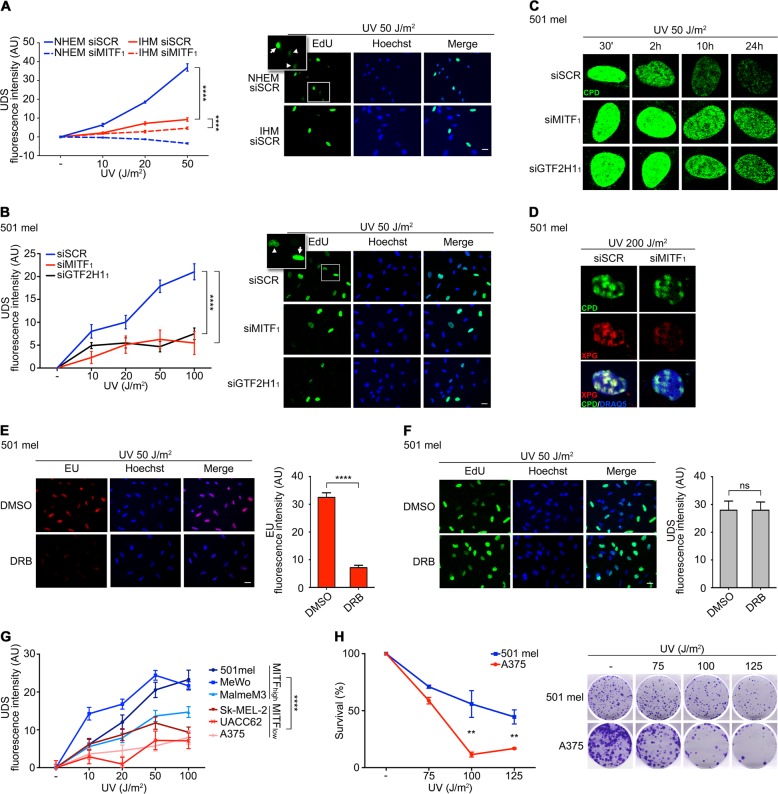
MITF–GTF2H1 axis augments NER and increases clonogenic survival after UVR. **a** UV-induced unscheduled DNA synthesis (UDS) in NHEM vs. IHM measured by EdU incorporation. **b** UV-induced UDS in 501 mel cells after siSCR, siMITF_1_ or siGTF2H1_1_ transfection. In **a** and **b**, graphs indicate mean±SEM of fluorescence intensity in ≥150 non-replicating nuclei for each UV-dosage. Right panels show representative micrographs of the corresponding irradiated cells at 50 J/m^2^. Insets: arrowhead depicts UDS; arrow depicts replicating cell; scale: 25μm. **c** Analysis of cyclobutane pyrimidine dimer (CPD) repair in UVB irradiated 501 mel cells after siSCR, siMITF_1_ or siGTF2H1_1_ transfection using immunofluorescence (IF) microscopy. **d** IF analysis of CPD and recruitment of XPG protein to sites of DNA damage induced by localized nuclear UVC irradiation after transfection of siMITF_1_ vs. siSCR. DRAQ5 used for nuclear counterstaining. **e** Analysis of transcriptional activity of 501 mel cells after 1-h DRB pulse treatment vs. DMSO control by measurement of EU incorporation. Graphs indicate mean ± SEM of fluorescence intensity in ≥100 nuclei. Depicted pictures were taken 6 h after UVR (scale: 25 µm). **f** UV-induced UDS assay in 501 mel cells in analogy to **e**. Graphs indicate mean ± SEM of fluorescence intensity in ≥100 nuclei (scale: 25 µm). **g** UV-induced UDS assay in melanoma cells with low vs. high abundance of MITF. Graphs indicate mean ± SEM of fluorescence intensity in ≥100 non-replicating nuclei. **h** Clonogenic cell survival assay of UV-irradiated MITF-abundant 501 mel cells vs. MITF-negative A375 cells. Graph represents mean ± SD from technical triplicates. **a**–**h** Experiments were done at least twice with similar results. All graphs were analyzed with a two-tailed unpaired *t*-test. **, *p* < 0.01, ****, *p* < 0.0001, n.s. not significant

Given MITF’s DNA damage responsiveness and control of GTF2H1 we sought to evaluate whether MITF activity impacts global NER capacity. To this end, we quantitatively assessed NER as a function of MITF or GTF2H1 by UV dosage-dependent unscheduled DNA synthesis (UDS) in primary melanocytes and melanoma cells utilizing a 5-ethylene-2ʹ-deoxyuridine (EdU) incorporation assay [38], which revealed compromised repair rates under MITF as well as GTF2H1 knockdown. In addition, MITF-high primary melanocytes showed a significantly greater unscheduled DNA synthesis than immortalized MITF-low melanocytes (Fig. 3a, b and Supplementary Figure 4a). Accordingly, repair of cyclobutane pyrimidine dimers (CPD) as a predominant type of UV-induced DNA lesion as well as cisplatin-induced primary DNA mono-adducts and subsequently formed intrastrand crosslinks, both of which constitute NER substrates, was substantially delayed upon depletion of MITF or GTF2H1 in primary melanocytes and melanoma cells, respectively (Fig. 3c, and Supplementary Figures 4b-d) [39]. The essential 3ʹ-endonuclease XPG, which physically interacts with GTF2H1 via a pleckstrin homology domain [28], was not recruited to DNA lesions after focal UVC-irradiation of MITF-depleted nuclei indicating a disassembly of NER complexes (Fig. 3d). Although NER was shown to be transcription-independent by simultaneous measurement of EU and EdU incorporation following UVR (50 J/m^2^) and 1-h pulse treatment with the CDK9 inhibitor 5,6-Dichloro-1-β-d-ribofuranosylbenzimidazole (DRB) in the presence of MITF, secondary transcriptional effects of MITF or GTF2H1 depletion on NER factors cannot be ruled out with certainty (Fig. 3e, f). Hence, we sought to assess NER in transcriptionally homeostatic melanoma cells with different expression levels of constitutive MITF. In a complementary gain-of-function experiment, we measured unscheduled DNA-synthesis after UVR upon retroviral expression of low-copy number MITF vs. empty vector control in MITF-negative A375 cells. In accordance with the observed reduction in UDS upon MITF and GTF2H1-directed RNA interference, we observed a strong positive association between the level of MITF expression and NER activity, which concurred with increased survival and colony formation of UV-irradiated MITF-abundant melanoma cells (Fig. 3g, h and Supplementary Figures 4e and f). On a MITF-negative background, low-copy-number retrovirus-mediated expression of GTF2H1 alone enhanced NER in a similar manner as observed under virus-driven expression of MITF emphasizing the importance of an MITF-dependent regulation of GTF2H1 in DNA repair (Supplementary Figure 4g).

### Modulation of GTF2H1 affects growth of the melanocytic lineage

**Fig. 4:**
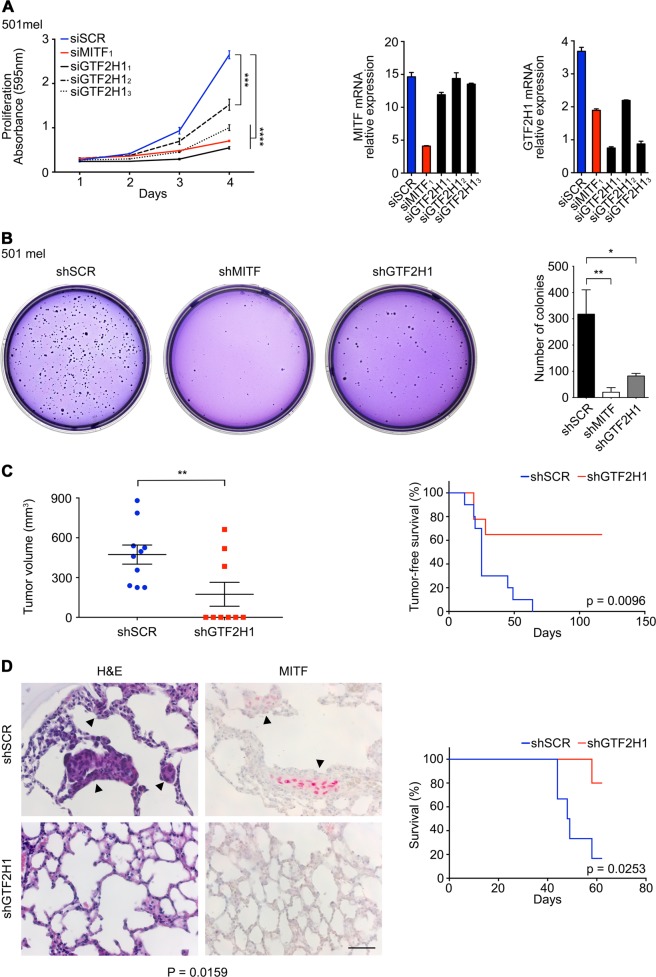
Disruption of MITF–GTF2H1 axis represses melanoma growth. **a** Proliferation of 501 mel cells after transfection with siMITF_1_ or three different siGTF2H1_1–3_ vs. siSCR. Graphs represent mean ± SD of crystal violet (CV) absorbance from technical triplicates (two-tailed unpaired *t*-test). Right panels, mRNA expression of MITF and GTF2H1 under RNAi as indicated. **b** Colony formation assay of transduced 501 mel cells expressing shRNA directed against MITF or GTF2H1 compared to shSCR control. Right panel, bars represent mean ± SD mean of the number of colonies from technical triplicates (two-tailed unpaired *t*-test). **a**, **b** Biological replicates revealed similar results. **c** End point analysis of tumor growth in shGTF2H1 vs. shSCR expressing melanoma xenografts after subcutaneous transplantation into SCID mice. Tumor growth in shGTF2H1 cohort (*n* = 3 of 9 animals) was associated with insufficient repression of GTF2H1 determined by expression analysis in FACS-sorted GFP-positive tumor cells (one-tailed unpaired *t*-test). Right panel, Kaplan–Meier analysis of tumor-free survival. shGTF2H1 cohort, *n* = 9 mice; shSCR cohort, *n* = 10 mice. **d** Immunohistochemical analysis exhibiting pulmonary spread of genetically modified shGTF2H1 vs. shSCR expressing melanoma cells after tail vein injection. H&E, hematoxylin & eosin staining; MITF, C5 monoclonal antibody (red signal). Arrowheads, melanoma cell infiltration. Right panel, Kaplan–Meier analysis of overall survival. shGTF2H1 cohort, *n* = 5 mice; shSCR cohort, *n* = 6 mice. *P*, log-rank test

To assess whether depletion of GTF2H1 recapitulates the senescence-like phenotype induced by knockdown of MITF, we utilized several independent siRNAs directed against GTF2H1, which caused a significant growth inhibition in vitro in a variety of melanoma cell lines (Fig. 4a and Supplementary Figures 5a-c). In long-term colony formation assays, lentiviral shRNA-mediated GTF2H1 or MITF depletion almost completely abrogated colony growth of 501 mel cells (Fig. 4b). In vivo, GTF2H1-directed RNA interference repressed growth and dissemination of 501 mel cells in subcutaneous and distantly spread xenograft mouse models resulting in significantly superior tumor-free and overall survival of the GTF2H1-knockdown cohorts compared to the control cohorts in Kaplan–Meier analyses (Fig. 4c, d).

**Fig. 5:**
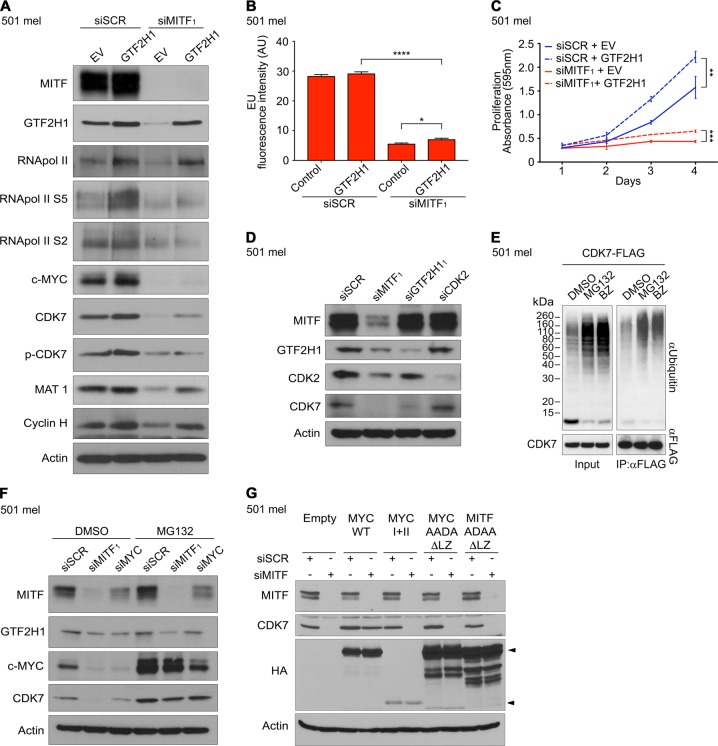
MITF depletion causes loss of MYC and TFIIH kinase. **a** Phospho-immunoblot analysis of RNA pol II, TFIIH-CAK, and MYC under forced expression of GTF2H1 in 501 mel cells and subsequent siMITF_1_ or siSCR transfection compared to EV control. **b** Transcriptional activity of 501 mel cells as measured by EU incorporation under conditions analogous to **a**. Graph indicates mean ±SEM of fluorescence intensity in ≥250 nuclei (two-tailed unpaired *t*-test). **c** Proliferation of 501 mel cells genetically modified analogous to **a**. Graphs represent mean ± SD of crystal violet absorbance from technical triplicates (two-tailed unpaired *t*-test). **d** Immunoblot analysis of 501 mel cells transfected with siSCR, siMITF_1_, siGTF2H1_1_ or siCDK2. **e** Analysis of CDK7 ubiquitination by immunoprecipitation of transfected FLAG-tagged CDK7 in 501 mel cells treated with 10 µM MG132 or 1 µM bortezomib (BZ) compared to DMSO control. Left panel, ubiquitination input control; right panel, FLAG-directed immunoprecipitation of ubiquitinated CDK7 using anti-ubiquitin antibody. Detection of CDK7-FLAG with anti-FLAG antibody as loading control. **f** Immunoblot analysis of 501 mel cells transfected with siSCR, siMITF_1_ or siMYC under proteasome inhibition with 10 μM MG132 vs. DMSO control. **g** Immunoblot analysis of CDK7 expression under MITF depletion vs. control in 501 mel cells upon expression of c-MYC wild type, MYC box I and II, and transcription- and dimerization-deficient mutants of c-MYC and MITF. Leucine zipper was deleted to prevent dominant-negative effects on wild-type MAX or MITF by sequestration. Upper arrowhead: MYC WT, MYC AADA ΔLZ and MITF ADAA ΔLZ; lower arrowhead: MYC I + II. Actin used as loading control. **a**–**g** Experiments were performed twice independently with very similar results

In primary human cutaneous melanomas, GTF2H1 expression positively correlated with clinical stage such as tumor thickness and depth of invasion indicating an association with tumor progression (Supplementary Figures 5d and e). In line with this notion, virus-driven overexpression of GTF2H1 enhanced CAK (TFIIH kinase) and RNA pol II activities as demonstrated by phosphorylation of CDK7 at threonine 170 and RNA pol II carboxy-terminal domain (CTD) at serine 5 and 2 enhancing RNA polymerase processivity at transcribed genes (Fig. 5a and Supplementary Figure 5f) [40]. In addition, forced expression of GTF2H1 was associated with an increase in proliferation of IHM and melanoma cells exhibiting a complete lack or low abundance of MITF, respectively (Supplementary Figures 5g-i).

### MITF depletion causes loss of c-MYC and TFIIH kinase

By contrast, under experimental depletion of MITF in 501 mel cells recombinant GTF2H1 rescued only minimal transcriptional activity and cellular growth as shown by loss of phosphoactivities of RNA pol II CTD and CDK7, diminished EU incorporation, and minimal increase in proliferation (Fig. 5a–c and Supplementary Figure 6a). Strikingly, repression of MITF caused a substantial loss of MYC and CDK7 the latter of which was barely rescued by GTF2H1 (Fig. 5a). Several independent siMITF_1–3_ almost completely abrogated MYC mRNA expression, whereas CDK7 transcripts were reduced by ∼2-fold (Supplementary Figure 6b). MITF-directed RNAi did not affect c-MYC mRNA expression in MITF-negative melanoma (A375) and non-melanocytic tumor cells (MCF7 breast cancer and U2OS osteosarcoma cells) in contrast to MITF-dependent 501 mel cells ruling out siRNA-mediated off-target effects on c-MYC (Supplementary Figure 6c). RNAi-mediated repression of MITF resulted in the strongest decrease in general transcriptional activity compared to MYC, GTF2H1 or combined MYC/GTF2H1-directed RNA interference, while MITF expression was not substantially affected by MYC or GTF2H1 depletion, corroborating a dominant role of MITF in transcriptional homeostasis of the melanocytic lineage (Supplementary Figures 6d and e).

To further explore the loss of CDK7 mechanistically, we first focused on posttranslational modifications leading to destabilization of CDK7. To this end, we depleted the direct MITF target CDK2, which has been shown to phosphorylate CDK7 on Thr170 and Ser164 in vitro as a potentially activating feedback loop, without discernible effect on CDK7 expression (Fig. 5d) [41]. Under the hypothesis that CDK7 is targeted for proteasomal degradation we utilized the proteasome inhibitors MG132 or bortezomib, both of which caused a marked increase in FLAG-tagged CDK7-ubiquitin conjugates immunoprecipitated from 501 mel cells (Fig. 5e). Accordingly, MG132 treatment of melanoma cells caused an accumulation of CDK7 and rescued its expression under experimental depletion of MITF or c-MYC, the latter of which is an established target of several ubiquitin ligases (Fig. 5f) [42]. As anticipated, proteasome inhibition resulted in a substantial accumulation of c-MYC in the presence and upon depletion of MITF as well as MYC-directed RNAi (Fig. 5f).

As a next step, we asked whether MITF or c-MYC could stabilize CDK7 through a posttranscriptional mechanism mediated by protein–protein interaction. To this end, we engineered a series of wild type and deletion mutant HA-tagged MITF and FLAG-tagged CDK7 fusion proteins for eukaryotic expression. In addition, we generated GST-CDK7 and wild type and deletion mutant FLAG-tagged MITF or c-MYC fusion proteins for prokaryotic expression. Immunoprecipitation experiments showed binding of recombinant CDK7-FLAG to wild type and DNA-binding-domain (DBD)-mutated MITF (MITF-HA R215Δ) (Supplementary Figure 7a). The interaction of MITF and CDK7 was independently confirmed utilizing GST-CDK7 (Supplementary Figure 7b). Further mutagenesis of MITF revealed binding of N- and C-terminal domains of MITF to GST-CDK7 (Supplementary Figure 7c). In similar experiments, we employed wild type and FLAG-tagged c-MYC deletion mutants which demonstrated binding of MYC box II transactivation domain to CDK7-GST (Supplementary Figures 7d and e). However, in rescue experiments utilizing a MYC wild type expression construct in comparison to transcription- and dimerization-deficient mutants of MITF or MYC with intact CDK7-binding domains, CDK7 was only preserved upon expression of transcription-competent endogenous MITF or recombinant wild type c-MYC (Fig. 5g). This finding clearly indicates a transcription-dependent regulation of CDK7 by MITF or MYC without discernible impact of the observed protein–protein interaction on CDK7 stability.

### MYC rescues general transcription in the absence of MITF

**Fig. 6:**
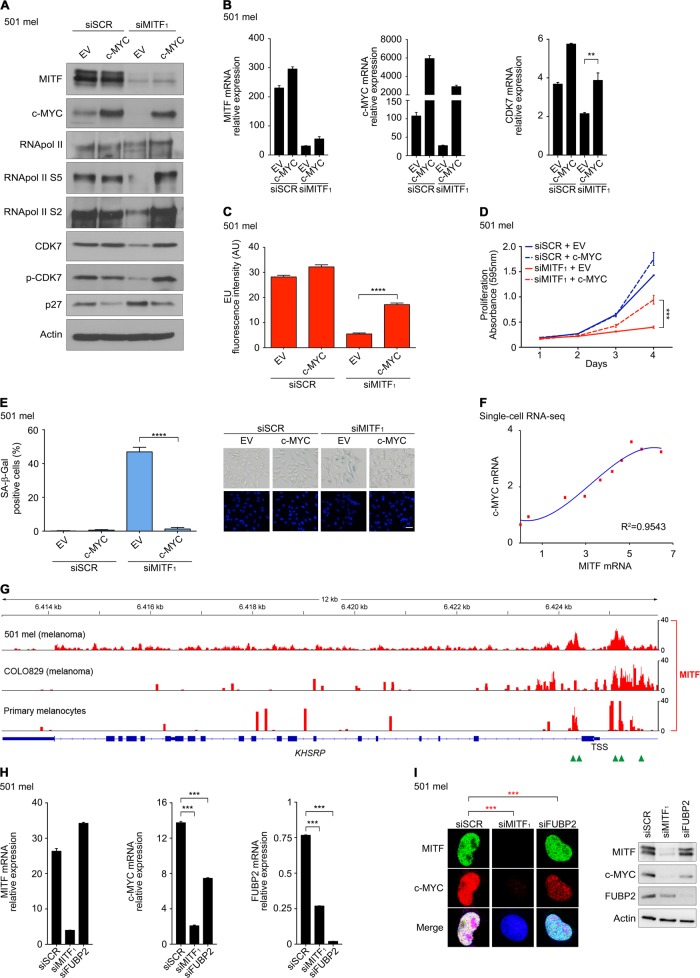
c-MYC rescues general transcription and prevents senescence in the absence of MITF. **a** Immunoblot analysis of 501 mel cells under retrovirus-driven c-MYC expression compared to EV and subsequent siMITF_1_ vs. siSCR transfection. **b** MITF, c-MYC, and CDK7 mRNA expression in 501 mel cells under conditions analogous to **a**. Relative expression was measured by qRT-PCR, normalized to GAPDH and given as mean ± SD from technical triplicates (two-tailed unpaired *t*-test). **c** Transcriptional activity measured as EU incorporation in 501 mel cells in analogy to **a** and **b**. Graphs indicate mean ±SEM of fluorescence intensity in ≥250 nuclei (two-tailed unpaired *t*-test). **d** Proliferation of 501 mel cells in analogy to **a**–**c**. Graphs represent mean ± SD of crystal violet absorbance from technical triplicates (two-tailed unpaired *t*-test). **e** SA-β-gal positivity in 501 mel cells at day 3 in analogy to **d**. Data represent mean ± SD from technical triplicates (two-tailed unpaired *t*-test). Micrographs display SA-β-gal signals and Hoechst 33342 nuclear staining under corresponding conditions (scale: 50 µm). **f** Regression analysis of MITF and MYC single-cell mRNA expression from a panel of metastatic melanomas (GSE72056). **g** ChIP‐seq tracks of MITF-binding signals at *FUBP2/KHSRP* gene locus in primary melanocytes, and 501 mel and COLO829 BT168F melanoma cell lines. Green arrowheads indicate MITF-binding consensus sequences (E box) at the promoter and first intronic region of the *FUBP2/KHSRP* gene. GEO accession numbers are listed under Materials and Methods. **h** MITF, c-MYC, and FUBP2 mRNA expression in 501 mel cells after siSCR, siMITF_1_, or siFUBP2 transfection. Relative expression was measured by qRT-PCR, normalized to GAPDH and given as mean ± SD from technical triplicates (two-tailed unpaired *t*-test). **i** Immunofluorescence and immunoblot analyses of MITF, MYC, and FUBP2 protein expression in analogy to **h**. DRAQ5 used for nuclear staining and actin used as loading control (two-tailed unpaired *t*-test of c-MYC fluorescence intensity in 100 nuclei is indicated). **a**–**e**, **h**, **i** Experiments were repeated twice with comparable results

In accordance with the aforementioned, retroviral expression of c-MYC preserved CDK7 at mRNA and protein levels, restored transcriptional activity, and prevented a senescence-like phenotype as shown by reduced β-galactosidase staining and p27 downregulation (Fig. 6a–e). As predicted, c-MYC was unable to maintain transcription of melanocyte-restricted MITF target genes resulting in de-differentiation as demonstrated for PMEL, MLANA, and TYRP1 pigmentation genes (Supplementary Figure 8a). Since the relationship between MITF and c-MYC had not been clearly defined yet, we analyzed accessible single-cell RNA-seq data from a panel of metastatic melanomas, which showed a strong positive correlation between the expression of MITF and c-MYC transcripts in individual cells of a given heterogeneous tumor cell population and across various tumor specimens (Fig. 6f) (GSE72056) [16]. The concordant expression of MITF and c-MYC transcripts was confirmed in a panel of genetically heterogeneous melanoma lines by RT-PCR (Supplementary Figure 8b). Notably, at the protein level MYC appeared to be decoupled from MITF in ∼15–20% of metastatic human melanoma samples (*n* = 18) and melanoma cell lines (*n* = 7) reflected by a low MITF/MYC expression ratio in whole cell lysates as demonstrated in immunoblot analyses (Supplementary Figures 8c and d).

Previously, we identified fuse binding protein 2 (FUBP2) as a potential MITF-target gene, which belongs to the family of far upstream element binding factors implicated in pulse-regulation of c-MYC through TFIIH [1, 43]. Hence, we hypothesized that MITF-dependent regulation of FUBP2 might at least partially affect MYC expression. In agreement with our hypothesis, in silico ChIP-seq analyses identified occupancy of MITF at proximal E box containing promoter sequences of *FUBP2* (*KHSRP*) in primary melanocytes and melanoma cell lines (Fig. 6g). MITF depletion resulted in isoform-specific downregulation of FUBP2 mRNA and protein. Repression of FUBP2 in turn caused a moderate reduction in MYC mRNA and protein expression compatible with a lack of pulse-regulation (Fig. 6h, i and Supplementary Figure 8e).

### TFIIH kinase is transactivated by MITF and MYC in the melanocytic lineage

**Fig. 7:**
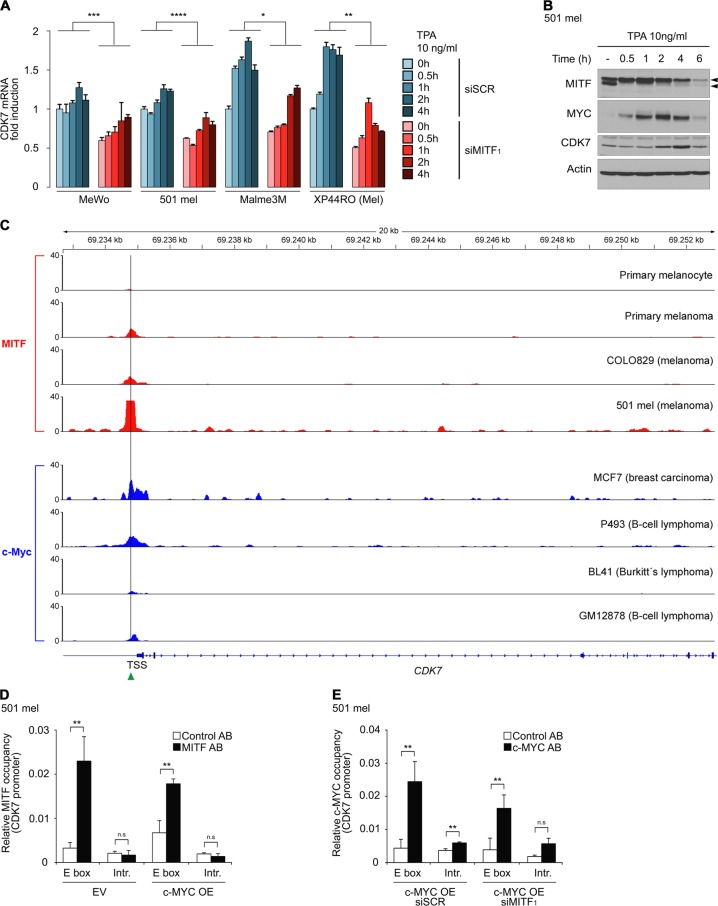
CDK7 is a direct transcriptional target of MITF and c-MYC. **a** Time course of CDK7 mRNA expression in 501 mel cells transfected with siMITF_1_ vs. control siRNA under treatment with phorbol ester (TPA). Data represent mean ± SD from technical triplicates (two-tailed paired *t*-test). **b** Immunoblot analysis of MITF, c-MYC and CDK7 protein expression in 501 mel cells under TPA treatment. Lower and upper arrowheads indicate non-phosphorylated (54 kd) and phosphorylated (60 kd) MITF forms, respectively. Actin used as loading control. **c** ChIP-seq tracks of MITF (marked red) and c-MYC (marked blue) binding signals at *CDK7* gene locus in primary melanocytes and primary melanomas, melanoma cell lines and non-melanocytic cancer cells, respectively. Green arrowhead indicates E box consensus sequence −86 base pairs upstream of the transcriptional start site of CDK7. GEO accession numbers of corresponding data sets are listed under Materials and methods. **d** ChIP, in vivo occupancy of MITF at CDK7 promoter in 501 mel cells in the presence or absence of forced c-MYC expression (OE overexpression) compared to empty vector (EV) including intron sequence and IgG controls. Mean ± SD from technical triplicates. **e** ChIP, in vivo occupancy of exogenous c-MYC (OE) at CDK7 promoter in 501 mel cells under siSCR or siMITF_1_ transfection. Intron sequence and IgG were used as controls. Mean ± SD is presented from technical triplicates. **d**, **e** Two-tailed paired *t*-test. **a**, **b**, **d**, **e** Experiments were performed twice and biological replicates revealed very similar results

To explore the regulatory mechanism of CDK7 transcription, we first treated several MITF-dependent melanoma lines under MITF-directed RNAi with 12-*O*-tetradecanoylphorbol-13-acetate (TPA), a potent activator of protein kinase C. TPA leads to activation of MAP kinase and subsequent phosphorylation of MITF S73 which mediates upregulation of transcriptional activity of MITF [33]. Across all melanoma lines tested, depletion of MITF was associated with a significant decrease in CDK7 transcription over time. Successful activation of MITF by TPA treatment was confirmed by immunoblot analysis which demonstrated a rapid shift of MITF protein to the slower migrating form (∼60kd) followed by consecutive upregulation of MYC and CDK7 (Fig. 7a, b). Next, in silico ChIP-seq analyses revealed binding of MITF to an E box (tCACGTG) consensus sequence −86 base pairs upstream of the transcriptional start site of CDK7 in primary melanocytes, primary melanoma and melanoma cell lines. In analogy, we identified MYC enrichment at the identical site in the CDK7 promoter in various ChIP-seq data sets from breast cancer and B-cell malignancies suggesting that both MITF and MYC are able to directly transactivate CDK7 (Fig. 7c). To provide evidence that MITF and MYC occupy the CDK7 promoter in the melanocytic lineage, we performed ChIP-experiments in MYC-overexpressing vs. empty vector control expressing 501 mel cells confirming enrichment of both MITF and MYC at the E box sequence in the CDK7 promoter (Fig. 7d, e). Interestingly, endogenous MITF also bound the CDK7 promoter under conditions of c-MYC overexpression (Fig. 7d). Likewise, c-MYC bound the CDK7 promoter in the presence of MITF, but also under conditions of MITF depletion (Fig. 7e). In regard to simultaneous presence of MITF and MYC at a given CDK7 promoter, these findings could implicate higher affinity binding of MITF but MYC might be able to replace gradually decreasing MITF in the regulation of CDK7.

## Discussion

TFIIH is a major effector of global genome repair as well as transcription-coupled repair (TCR) [44]. By direct regulation of GTF2H1 MITF controls both NER pathways as demonstrated here by an augmented unscheduled DNA synthesis and recovery of RNA synthesis after UVR utilizing 5-ethynyl-2’-uridine incorporation as a surrogate of TCR under conditions involving highly abundant MITF. In addition, the observed loss of CDK7 under experimental depletion of MITF abrogates the restart of transcription after completion of repair [22]. MITF’s impact on global transcription superimposes over TCR and prevents a formal discrimination between direct and indirect MITF-dependent effects on strand-specific repair, but RNAi-mediated repression of the TCR-specific helicase Cockayne syndrome protein B (CSB) in melanoma cells recapitulated the NER phenotype under MITF depletion associated with a marked decrease in unscheduled DNA synthesis <12 h after UVR (Supplementary Figure 9) [45]. The experimental evidence presented here primarily pinpoints GTF2H1 as a transcriptional target of MITF activity accounting for the observed NER as well as general transcription phenotypes modulated by MITF, however additional targets operating at the interface of repair and transcription could be involved in particular with regard to TCR.

Within a given tumor cell population the dichotomous behavior of MITF shuttling between low and high expression levels is largely enigmatic, but it may reflect invasive or dormant vs. proliferative states with varying transcriptional demands [14]. Invasive behavior has been associated with high expression of AXL and therapy resistance [15, 16]. However, a proliferative state is essential to primary tumor progression and macro-metastatic manifestation. In this regard, it has been unclear how melanoma cells with low MITF survive and proliferate in vivo, the majority of which apparently do not exhibit elevated AXL protein expression [16]. In contrast to the correlative association between MITF and c-MYC in single-cell RNA-seq data sets on metastatic melanoma, preliminary data indicate that expression of c-MYC appears to be decoupled from MITF at the protein level in bulk MITF-low tumor cells and TERT-immortalized melanocytes (Supplementary Figures 8c and d). We propose that a gradual loss of MITF as sometimes observed during melanoma progression might be supplanted by a variable decrease in MYC turnover rather than transcriptional upregulation allowing for accumulation of MYC protein, which preserves CDK7 and acts as a compensatory switch mechanism to maintain transcriptional homeostasis and to modulate proliferative activity at the expense of melanocyte-specific gene activation (Fig. 6a–e and Supplementary Figure 8a). In accordance, c-MYC has previously been implicated in suppression of BRAF^V600E^-induced senescence in melanocytes and melanoma progression, while loss of CDK7 results in senescence [46, 47]. Transcriptional downregulation of MYC might account for the resistance of melanoma cells toward BET bromodomain inhibition by the small molecule JQ1, which has been shown to downregulate MYC transcription and to disrupt the MYC-dependent transcriptional program in multiple myeloma, resulting in potent antiproliferative effects as opposed to the melanoma models examined in this study (Supplementary Figure 8f) [48]. Nevertheless, transcriptional re-wiring of c-MYC has recently been described in BRAF-inhibitor resistant melanoma sensitizing to bromodomain inhibition [49]. However the MITF/MYC ratio may be, the transcription dependency of melanoma cells is not significantly altered by a gradual decrease in MITF expression and concomitant de-differentiation as reflected by similar sensitivities of melanoma cells with low or high abundance of MITF toward the covalent CDK7 inhibitor THZ1. Our studies identify c-MYC as a surrogate of MITF which directly activates CDK7 likely accounting for the non-selective efficacy of THZ1 as observed here and previously by others in the context of super-enhancer-driven oncogenesis (Supplementary Figures 8g and h) [50, 51].

**Fig. 8:**
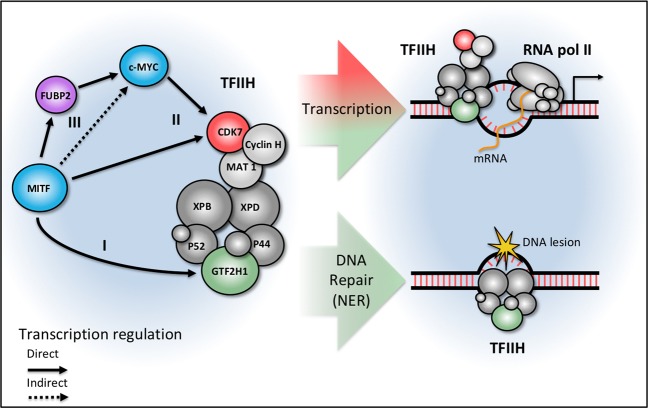
Model of TFIIH-CAK regulation by MITF and c-MYC. In the melanocytic lineage, the microphthalmia-associated transcription factor (MITF) determines transcriptional homeostasis and genomic integrity through regulation of the transcription factor II H (TFIIH) and the CDK-activating kinase (CAK) complex. (I) MITF controls general transcription and UVR-induced nucleotide excision repair by targeting the transcription initiation complex through direct transactivation of GTF2H1 as a core element of TFIIH. (II) The melanocyte master regulator MITF and its structural homolog c-MYC control the expression of CDK7, the catalytic subunit of CAK complex, which has a dual role in transcription and cell cycle activities. (III) MITF controls MYC activities by transactivation of fuse binding protein (FUBP2) involved in pulse regulation of MYC. In addition, MYC is likely affected by MITF-dependent effects on proliferation

Together, our findings place MITF as a safeguard into the UVR-induced DNA-damage response pathway activating NER and transcription, which represent a significant conceptual advance in our understanding of global transcriptional regulation in the melanocytic lineage under genotoxic stress (Fig. 8). Moreover, the dependency of TFIIH-CAK on the melanocyte master regulator MITF or its structural homolog c-MYC extends the conceptional idea of a CDK7-targeted treatment approach to melanoma far beyond super-enhancers as determinants of oncogenesis.

## Materials and methods

### Primary human materials

Primary human melanocytes were obtained from the Tissue Resource Core of Yale SPORE in Skin Cancer (New Haven, CT, USA) or LONZA (Walkersville, MD, USA). Primary cutaneous and metastatic melanoma samples were collected after written informed consent at the Department of Dermatology and Venerology, University Medical Center Hamburg-Eppendorf, Germany. Studies on primary human materials have been approved by the Institutional Ethics Committee of the City of Hamburg (OB-073-04 and MC-028/08). Clinico-pathological characteristics are presented in Supplementary Table S2.

### Cell culture

Human telomerase reverse transcriptase (hTERT)—immortalized human melanocytes (IHM) were obtained from ABM (Richmond, BC, Canada). Human A375, Malme3M, SK-MEL-2, WM164, UACC62, MeWo, murine B16V melanoma cells, human breast carcinoma MCF7 cells, human osteosarcoma U2OS cells, NCI-H69 small cell lung cancer and HEK 293T cells were obtained from ATCC or DSMZ (Braunschweig, Germany). U87MG human glioblastoma cells, XP44RO (Mel) and 501 mel melanoma cell were generously provided by Dr. Cecile Maire (Hamburg, Germany), Dr. Hoeijmarker (Rotterdam, the Netherlands), and Dr. Ruth Halaban (Tissue Resource Core of Yale SPORE in Skin Cancer, New Haven, CT, USA), respectively. All cell lines were maintained in Dulbecco´s modified Eagle´s media (GIBCO) with 10% FBS except for 501 mel and XP44RO (Mel) cells, which were cultivated in Ham’s F-10 Nut Mix media (GIBCO) with 10% FBS. Primary human melanocytes were cultured in MCDB153 media (Sigma) with 4% FCS, 1% penicillin/streptomycin, 5 mg/ml insulin, 1 mg/ml transferrin, 0.6 ng/ml human basic FGF, 10 ng/ml TPA and 13 mg/ml BPE or TICVA media containing Ham’s F10, 7% FBS, 1% penicillin/streptomycin, 2 mM glutamine, 50 ng/ml TPA, 0.1 mM IBMX, Na_3_VO_4_, and 1 mM dbcAMP. IHM were cultured in PriGrow II medium (ABM) with 10% FBS. Cells were grown at 37 °C and 5% CO_2_. Authenticity of human cell lines has been confirmed by STR analysis. Species of animal lines was confirmed by Cytochrome-C Oxidase I DNA barcoding (DSMZ, Braunschweig). Cell lines were free of mycoplasma contamination.

### Inhibitors and drugs

Cisplatin (Neocorp) was given as indicated. To inhibit transcription, 5,6-dichloro-1-beta-D-ribofuranosylbenzimidazole (DRB, Sigma Aldrich) (100 µM) was added to the media 1 h before UVR. Forskolin (Sigma Aldrich) was given at a concentration of 20 µM 4 h before read-out. MG132 (Calbiochem) or bortezomib (Cell Signaling) were applied for 4–18 h at concentrations of 10 or 1 µM, respectively. 12-*O*-tetradecanoylphorbol 13-acetate (TPA, Sigma Aldrich) (10 ng/ml) or recombinant human stem cell factor (SCF, PeproTech) (20 ng/ml) were added to the cells after overnight serum starvation. The covalent phenylaminopyrimidine inhibitor of CDK7 (THZ1, APExBio) or the BET inhibitor (JQ1, Cayman Chemical) were administered at the indicated concentrations and viability of cells was quantified using WST-1 (Roche) assay at 48 and 72 h.

### Real-time RT-PCR analysis

Total RNA was isolated using Trizol (Invitrogen) or RNeasy Plus Mini Kit (Qiagen) and converted into cDNA by reverse transcriptase (Invitrogen or Promega). cDNA expression was quantified using FastStart DNA Master^PLUS^ SYBR Green I Kit (Roche) on the Light Cycler 480 machine (Roche). Data were normalized to GAPDH or B2M expression for reproduction. Gene specific primer sets are available on request.

### Western blotting

Whole cell lysates were prepared in RIPA buffer and loaded onto SDS-PAGE. Western blotting was performed using following antibodies: anti-MITF C5 (Millipore, MAB3747), anti-human GTF2H1 (AbD, MCA4041Z), anti-c-Myc (Cell Signaling, #5605), anti-human CDK7 (Cell Signaling, #2916; Santa Cruz, sc-365075), anti-human phospho-CDK7 T170 (Abcam, ab155976), anti-human MAT-1 (Santa Cruz, sc-6234), anti-human cyclin H (Abcam, ab92376), anti-human CDK2 (Santa Cruz, sc-53220), anti-human RNA polymerase II (Bethyl, A300-653A), anti-human phospho-RNA polymerase II (S2) (Bethyl, A300-654A), anti-human phospho-RNA polymerase II (S5) (Bethyl, A300-655A), anti-human p27 (Santa Cruz, sc-528), anti-HA-tag HRP conjugate (Cell Signaling, #2999), anti-FLAG (Sigma Aldrich, F1804), and anti-β-Actin (Sigma Aldrich, A2228). Anti-mouse immunoglobulins/HRP (Dako, P0447) and anti-rabbit immunoglobulins/HRP (Dako, P0448) were used as secondary antibodies. Proteins were visualized with Amersham ECL solution (GE Healthcare, RPN2106).

### GST pull down

Full length versions and deletion mutants of human MITF, MYC, and CDK7 were expressed as GST-fusion proteins in AVB100 bacteria. For MITF and MYC, the GST-tag was removed by digestion with Prescission protease (GE Healthcare, 27-0843-01). For GST pull down, 12.5 µg of GST-CDK7 were bound to 10 µl of GSH-sepharose (GE Healthcare, 17075601) in the presence of protease inhibitors for 1 h at RT. Subsequently, beads were washed (2×) with PBS, blocked with 1% BSA in PBS for 30 min at RT, incubated for 1.5 h at 4 °C with 3–6 µg GST-free MITF and MYC, respectively, washed with PBS and eluted with 20 mM GSH (Sigma, 4251) in 100 mM Tris-HCl (pH 8.0) for Western blot analysis; free GST served as binding control.

### Cell transfection and RNA interference

For RNA interference cells were transfected with 24 nM of various siRNAs (sequences available upon request) using lipofectamine RNAiMAX (Invitrogen). Lipofectamine 2000 and 3000 (Invitrogen) were used for luciferase reporter assays and in vivo binding of MITF to CDK7 in 501 mel and HEK 293T, respectively. For overexpression of wild-type c-MYC, and MITF/c-MYC mutants, 501 mel cells were transfected with 1 µg/ml of DNA using lipofectamine 3000 (Invitrogen) followed by selection with geneticin (G418).

### Cloning

RNAi-resistant MITF mutant (MITF_RES_), MITF and MYC deletion mutants were generated by PCR cloning. MITF_RES_ was cloned as triple silent A/G point mutation (aa ERRRRF) into the retroviral vector pBABE-puro. Following deletion mutants were generated: MYC I + II (aa 1–180 including box I and II and transactivation domain), MYC-AADA-ΔLZ (mutation of the DNA-binding domain RQRR to ADAA, aa 379–382 and leucine zipper domain deletion, aa 428–454) and MITF-ADAA-ΔLZ (mutation of the DNA-binding domain RRRR to AADA, aa 214–217 and leucine zipper domain deletion, aa 267–288). Constructs were cloned into pCMV6-AC-IRES-GFP for transfection and transient expression.

### Adenovirus infection and RNA preparation

For adenoviral infection of human melanoma cells, recombinant AdEasy adenoviruses expressing HA-MITF-IRES-hrGFP (AdV-MITF), control peptide-IRES-hrGFP (AdV-control), and HA-MITF(DN)-IRES-hrGFP [AdV-MITF(DN)] were used as described before [52].

### UV exposure and detection of UV-induced cyclobutane pyrimidine dimers (CPD)

Cells were exposed to UVB (50 J/m^2^) in a CL-1000 Ultraviolet Crosslinker (UVP). For localized UV irradiation, cells were irradiated through an isopore-polycarbonate membrane (3-μm pore diameter) (Millipore) using UVC (200 J/m^2^) in a Stratalinker 1800 (Stratagene). For UV-induced CPDs anti-thymine dimer antibody KTM53 (Kamiya Biomedical Company, MC-062) was used followed by incubation with Alexa Fluor 488 donkey anti-mouse antibody (Invitrogen, A-21202). Nuclei were counterstained with DRAQ5 (Cell Signaling Technology, #4084). For XPG detection cells were stained with anti-XPG (Sigma Aldrich, X1629) and developed with Cy3-AffiniPure F(ab’)2 fragment goat anti-rabbit IgG (Jackson ImmunoResearch, 111–166–003). Fluorescence images were obtained with a ConfoCor 2 fluorescence microscope (Carl Zeiss Microscopy).

### UV-induced unscheduled DNA synthesis (UDS)

5-ethynyl-2ʹ-deoxyuridine (EdU) incorporation was used to quantify UDS. Cultured cells on coverslips were irradiated at different doses of UVB (10–100 J/m^2^) in a CL-1000 Ultraviolet Crosslinker (UVP). Subsequently, cells were incubated with serum-free media supplemented with 10 µM EdU and 1 µM of fluorodeoxyuridine (FdU) for 6 h. Cells were then fixed with 3.7 % paraformaldehyde, permeabilized with 0.5% triton X-100 and after washing with PBS acid extraction was performed using Bouin’s fixative to reduce background signals. Incorporated EdU was detected using the Click-iT EdU Imaging Kit (C10337, Invitrogen) following the manufacturer’s recommendations. Images were obtained using a fluorescence microscope (Leica DMIL). Fluorescence signal intensity was measured in ≥100 nuclei and expressed in arbitrary units (AU) using ImageJ software.

### Measurement of Pt-(GpG) adducts in DNA

Plated cells were treated with cisplatin (20 µg/ml) for 4 h and maintained in drug-free media for up to 48 h. Cell aliquots were harvested, immunostaining and analysis were performed as described before [53]. Mean values ± 95% confidence intervals of ≥100 cells per sample were calculated and expressed as arbitrary fluorescence units (AFU).

### Assessment of RNA synthesis

After UVR cells were incubated with 1 mM 5-ethynyl-uridine (EU) for 1 h and subsequently fixated with 3.7% formaldehyde in PBS for 15 min. EU incorporated into newly synthesized RNA was detected using the Click-iT RNA Alexa Fluor 594 Imaging Kit (Invitrogen) following the manufacturer’s recommendations. Images were obtained using a fluorescence microscope (Leica DMIL). Fluorescence signal intensity was measured in ≥100 nuclei and expressed in arbitrary units using ImageJ software. Total, mRNA and rRNA were measured in triplicates by spectrophotometry (NanoDrop) and fluorescence electropherogram on a Bioanalyzer instrument (Agilent) and normalized to cell numbers. To determine RNA content per cell, cells were stained using the metachromatic dye acridine orange (AO, Sigma Aldrich) at a concentration of 10 μg/ml and subsequently analyzed by flow cytometry on a BD FACS Canto.

### Gene expression array analysis

The gene expression profiles on melanoma tissues were downloaded from the Gene Expression Omnibus (GEO) database (GSE7553). Primary and metastatic melanoma samples (supplemented by melanoma in situ and epidermal normal melanocytes) were separated into two classes based on MITF expression. A sample was considered MITF-high, if it exhibited a signal greater than 4000 from the probe set 207233_s_at. Samples were sorted according to MITF expression level and sample type. Comparative Marker Selection analysis was carried out with GenePattern (http://genepattern.broadinstitute.org/gp) with 10,000 permutations. Probe sets with *P*-values < 0.05 are considered significant. Heat maps were generated with GenePattern Heatmap Viewer.

### GTF2H1 promoter mutagenesis and luciferase reporter assays

A fragment of the human GTF2H1 promoter (bp −882 to +159, relative to the transcription start site) was generated by PCR and cloned into the pGl-3 basic vector (Promega) upstream of the luciferase reporter gene. In addition, three E box sequences in the −882/+159 bp GTF2H1 promoter were mutated from CACGTG to GAGGTG using site-directed mutagenesis (Stratagene). GTF2H1 promoter constructs were co-transfected with pGL4.73 [*hRluc*/SV40] plasmids (Promega) upon serum withdrawal. A wild-type MITF pcDNA3.1 expression vector vs. control vector was co-transfected into A375 melanoma cells. Forskolin (Sigma Aldrich) was given as indicated above. To assess the responsiveness of GTF2H1 promoter after overexpression of wild-type MITF vs. SUMOylation-defective MITF, wild-type MITF or SUMOylation-defective MITF-E318K pCMX-PL2 expression vector vs. control vector was co-transfected into HEK 293T cells. Depending on the experimental context readout was performed 48 or 72 h after transfection using the Dual Luciferase Reporter Assay System (Promega) according to the manufacturer´s protocol.

### ChIP and ChIP-seq analysis

For chromatin immunoprecipitation (ChIP) analysis 2×10^7^ 501 mel cells were utilized for DNA-protein cross-linking with 1% formaldehyde. For immunoprecipitation, sheared chromatin equaling 1×10^6^ cells was incubated with 5 µg of either goat polyclonal anti-MITF (Santa Cruz, sc-11002X) or normal goat IgG (Santa Cruz, sc-2028) antibody for GTF2H1 promoter analysis. For CDK7 promoter analysis we used a mouse monoclonal anti-MITF (Active Motive, 39789) or a rabbit polyclonal anti-c-MYC (Cell Signaling, #9402) and isotype control mouse IgG1 (Biolegend, 401402) and rabbit IgG (Santa Cruz, sc-2027). Quantitative real-time PCR was applied on a Light Cycler instrument (Roche) to amplify DNA fragments containing E boxes in *GTF2H1* or *CDK7* promoter. *GTF2H1* intron 1 or the *CENPH* intron 1 were used as negative control loci (primer sequences are available on request). Following ChIP-seq datasets were downloaded from the GEO database and analyzed to obtain extended data on *FUBP2/KHSRP* and *CDK7* loci: Primary melanocytes (GSM1226221), primary melanoma (GSM1484317), COLO829 (GSM1226224), 501 mel (GSM1517751), MCF7 (GSM1006877), P493 (GSM1234501), BL41 (GSM762710), and GM12878 (GSM754334).

### Next-generation RNA sequencing

Next-generation RNA sequencing and spike-in normalization were done as previously described [35]. Library preparation was performed with NEBNext® Ultra™ RNA Library Prep Kit for Illumina® (NEB E7530) following manufacturer’s recommendations using 640 ng total RNA each. ERCC RNA spike-In reference sequences (Ambion, 4456739) were incorporated into the human reference assembly (Ensembl GRCh38.77). TopHat (v2.0.13) was used to align reads to resulting reference sequences and read counts were calculated with HTSeq (v0.6.1) [54, 55]. Subsequent processing was done with DESeq2 and R [56, 57]. For the assessment of total expression, data were normalized based on reads aligned to ERCC sequences. For measuring differential expression between siMITF and siGTF2H1, size factors were estimated by DeSeq2 without taking the ERCC sequences into account. RNA-seq data reported in this article have been deposited in the European Nucleotide Archive and are available under the accession code PRJEB30337.

### Proliferation, senescence, colony formation, and clonogenic cell survival assay

To measure proliferation, cells were seeded at low confluence in 24-well plates in triplicate and fixed in 3.7% paraformaldehyde on indicated days for subsequent staining with crystal violet (CV) 0.05%. After washing the content of cellular CV was extracted with acetic acid 10% and absorbance was measured at 595 nm. For growth-rescue experiments retroviral vector pBABE-puro-GTF2H1 or pBABE-puro-c-MYC vs. pBABE-puro-empty were employed. For quantitative assessment of senescence, 501 mel cells were transduced with different retroviral vectors (pBABE-puro-MITF_WT_, pBABE-puro-MITF_RES_, or pBABE-puro-MYC vs. pBABE-puro-empty) followed by puromycin selection and subsequent transfection with siMITF vs. siSCR RNA. Cells were seeded in 24-well plates in triplicate and kept in culture for three days. SA-β-gal staining was carried out using the senescence β-galactosidase staining Kit (Cell Signaling) following the manufacturer’s recommendations. For assessment of clonogenic growth in soft agar pLKO.1-puro-CMV-tGFP shRNA lentiviruses expressing scrambled, MITF or GTF2H1 shRNA were generated using HEK 293T cells co-transfected with compatible packaging plasmids. 501 mel cells were infected with lentivirus followed by puromycin selection. Transduced 501 mel cells were grown in 0.35% soft agar media for 30 days. The number of colonies per plate was quantified after staining with 0.05% crystal violet. To determine clonogenic cell survival, 501 mel and A375 cells were seeded in six-well plates (500, 750 or 1000 cells per well), and UVB-irradiated at the indicated doses. After irradiation cells were maintained in complete media at 37 °C and 5% CO_2_ for 12 days. Subsequently, cells were fixed with 3.7% paraformaldehyde and stained using 0.05% crystal violet for quantification of colonies. The percentage of survival was calculated relative to non-irradiated cells.

### TMA staining

Tissue microarrays (TMA) comprised *n* = 140 primary cutaneous melanomas (MM). Immunostaining of MITF, S-100 and GTF2H1 was done in 136 out of 140 eligible TMA-spotted melanoma samples. For immunohistochemistry (IHC) we used anti-human MITF (Dako, M3621) or anti-GTF2H1 (AbD, MCA4041Z) at a dilution of 1:100 for 40 min at RT or overnight at 4 °C. Permanent AP Red (Zytomed, ZUC001–125) was used for detection and slides were counterstained by hematoxylin. AP red signal frequencies and intensities were evaluated semi-quantitatively by assignment of an expression score (0, negative; >0 ≤ 1.5, low; >1.5 ≤ 2.5, intermediate; >2.5 high). IHC analyses were performed in a blinded fashion by two independent examiners. IHC staining of MITF was re-validated by immunofluorescence co-staining of MITF and S-100 for unambiguous detection of melanoma cells (Supplementary Figure 2c). Slides were incubated at 4 °C overnight with anti-human MITF (Dako, M3621) and anti-S-100 (Progen, 16100) antibodies at a dilution of 1:100 and 1:200, respectively. Anti-mouse Alexa 488-coupled and anti-rabbit Alexa 555-coupled (Molecular Probes Europe BV) were used as secondary antibodies. Nuclei were stained by DAPI. Isotype-specific antibodies were used as negative controls.

### Animal studies

All animal studies were performed according to ARRIVE guidelines and regulations of institutional animal care and use after approval by the Institutional Animal Care and Use Committee of the City of Hamburg (V1/591-00.33 No. 131/13). Severe combined immunodeficiency disorder (SCID) female 8- to 12-week-old mice (strain: *Prkdcscid*) (Charles River Laboratories) were used as allograft recipients. 501 mel cells were transduced with SCR-shRNA or GTF2H1-shRNA expressing lentiviruses (pLKO.1-puro-CMV-tGFP shRNA; Sigma-Aldrich). Viability of transduced cells (>90%) was verified at the day of transplantation. Gene-modified cells were either injected into subcutaneous tissue of the dorsolateral flank (2×10^6^ cells) or into the tail vein (1×10^6^ cells). Animals were not randomized with regard to treatment allocation. Two animals were excluded from further analyses (one animal each in subcutaneous and IV xenotransplantation experiments from GTF2H1 knockdown cohorts) due to fatal adverse events during general anesthesia. Subcutaneous tumors were measured manually in two diameters. Tumor volume was calculated according to the formula: (width^2^ × length)/2. Weight loss of ≥20% or ulceration after tumor engraftment was defined as stopping rule. End point was defined by tumor burden according to clinical assessment score or end of follow-up in the absence of tumor growth (day 117 after subcutaneous injection). Primary tumors and lung tissue were harvested and processed according to standard procedures. An approximate number of pulmonary (micro)metastases was determined by examining ten sections from the right lung of each animal separated by a minimum distance of 40 nm taken from the middle of each paraffin block. Pulmonary metastases were counted by two independent examiners; mean of ten sections was multiplied with the number of sections and correction factor 0.8 in order to estimate the total number of distantly spread cells per lung. Cell clusters consisting of ≤2 cells and intravascular cell clusters were excluded from the analysis.

### Statistical analyses

With regard to the tissue microarray Fisher’s exact test and chi-square-test were used to analyze frequency tables. Mann–Whitney Rank Sum test was used to compare groups of patients (25%/75% percentile) and Spearman correlation coefficient was calculated to describe the correlation of parameters. In mouse experiments, tumor-free as well as overall survival was calculated from the date of xenotransplantation to death. Distant spread of melanoma cells in IV xenotransplantation studies was assessed in a blinded fashion by two independent investigators. Kaplan–Meier analyses were performed to evaluate the association between survival and gene-modulating intervention. Log-rank tests were applied to assess statistical significance using Graph Pad Prism software. Sample size was estimated according to *N*_g,min_ = 2×[(*t*_2alpha_ + *t*_2beta_)/(*D*_rel/s_)]^2^ with P1 (alpha) = 0.05 and P2 (beta) = 0.2 to achieve a power of 80%. One-tailed unpaired Student’s *t*-test, two-tailed paired Student’s *t*-test or unpaired Student’s *t*-test was used to perform other statistical analyses of parametric data except for Cspl-induced Pt-(GpG) intrastrand adducts, which were tested for 95% CI. In general, distribution of data was evaluated for ≥100 parametric data points if available. Distribution/variance of data was evaluated using F-test. In case of unequal distribution non-parametric tests were applied such as Mann–Whitney *U*-test (two-tailed). Experimental samples/data were not excluded from the analyses except for two mice inoculated with shGTF2H1-expressing 501 mel cells which inadvertently died during general anesthesia.

## Supplementary information

Supplementary Figure 1

Supplementary Figure 2

Supplementary Figure 3

Supplementary Figure 4

Supplementary Figure 5

Supplementary Figure 6

Supplementary Figure 7

Supplementary Figure 8

Supplementary Figure 9

Supplementary Table 1

Supplementary Table 1
