# Supplementary material for: Lineage-specific control of TFIIH by MITF determines transcriptional homeostasis and DNA repair
Source: Oncogene. 2019 Jan 16;38(19):3616–35. doi: 10.1038/s41388-018-0661-x (PMC6756118; doi:10.1038/s41388-018-0661-x)
Supplement: Supplementary file 1 — Supplementary Figure 1 [file 41388_2018_661_MOESM1_ESM.pdf]

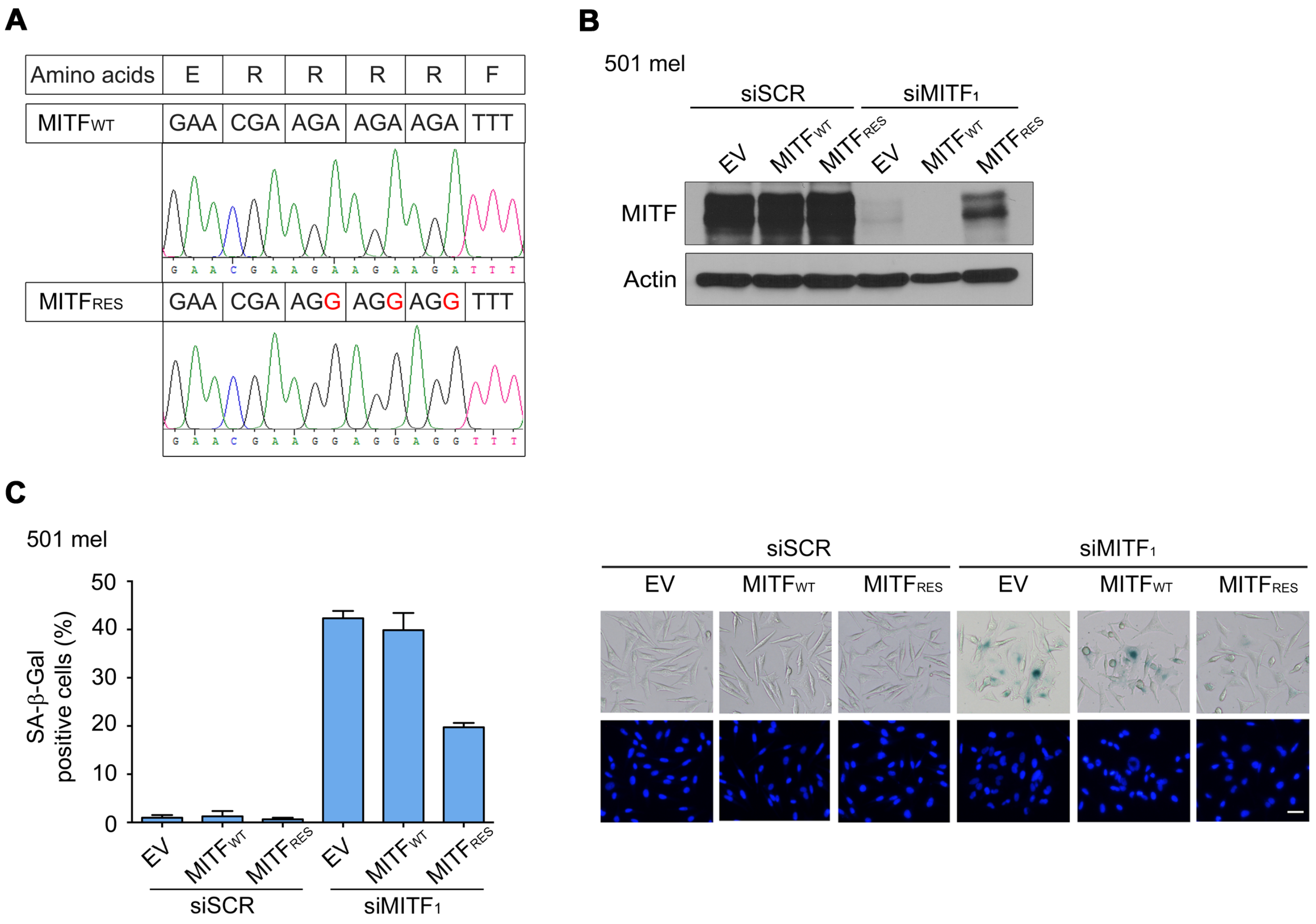

Supplementary Figure 1. RNAi resistant MITF mutant rescues senescence phenotype under MITF directed RNAi. **a**. Sequence chromatogram representing basic region of wildtype MITF ($MITF_{WT}$) and siRNA-resistant mutant MITF ($MITF_{RES}$); silent nucleotide exchanges are shown in red not affecting amino acid sequence presented above. **b**. Immunoblot analysis of MITF after retroviral expression of $MITF_{WT}$, $MITF_{RES}$ or empty vector (EV) under $siMITF_1$ vs. siSCR. Actin used as loading control. **c**. Left graph: Percentage of SA-β-gal-positive 501 mel cells after retroviral expression of $MITF_{WT}$, $MITF_{RES}$ or empty vector and subsequent siSCR or $siMITF_1$ transfection. Graph represents mean ±SD from technical triplicates. Right panel: Micrographs of representative images of senescence-associated β-galactosidase (SA-β-gal) staining under conditions analogous to B left panel. Hoechst 33342 (blue) used for nuclear counterstaining. Scale: 50 µm.
