# Supplementary material for: Lineage-specific control of TFIIH by MITF determines transcriptional homeostasis and DNA repair
Source: Oncogene. 2019 Jan 16;38(19):3616–35. doi: 10.1038/s41388-018-0661-x (PMC6756118; doi:10.1038/s41388-018-0661-x)
Supplement: Supplementary file 2 — Supplementary Figure 2 [file 41388_2018_661_MOESM2_ESM.pdf]

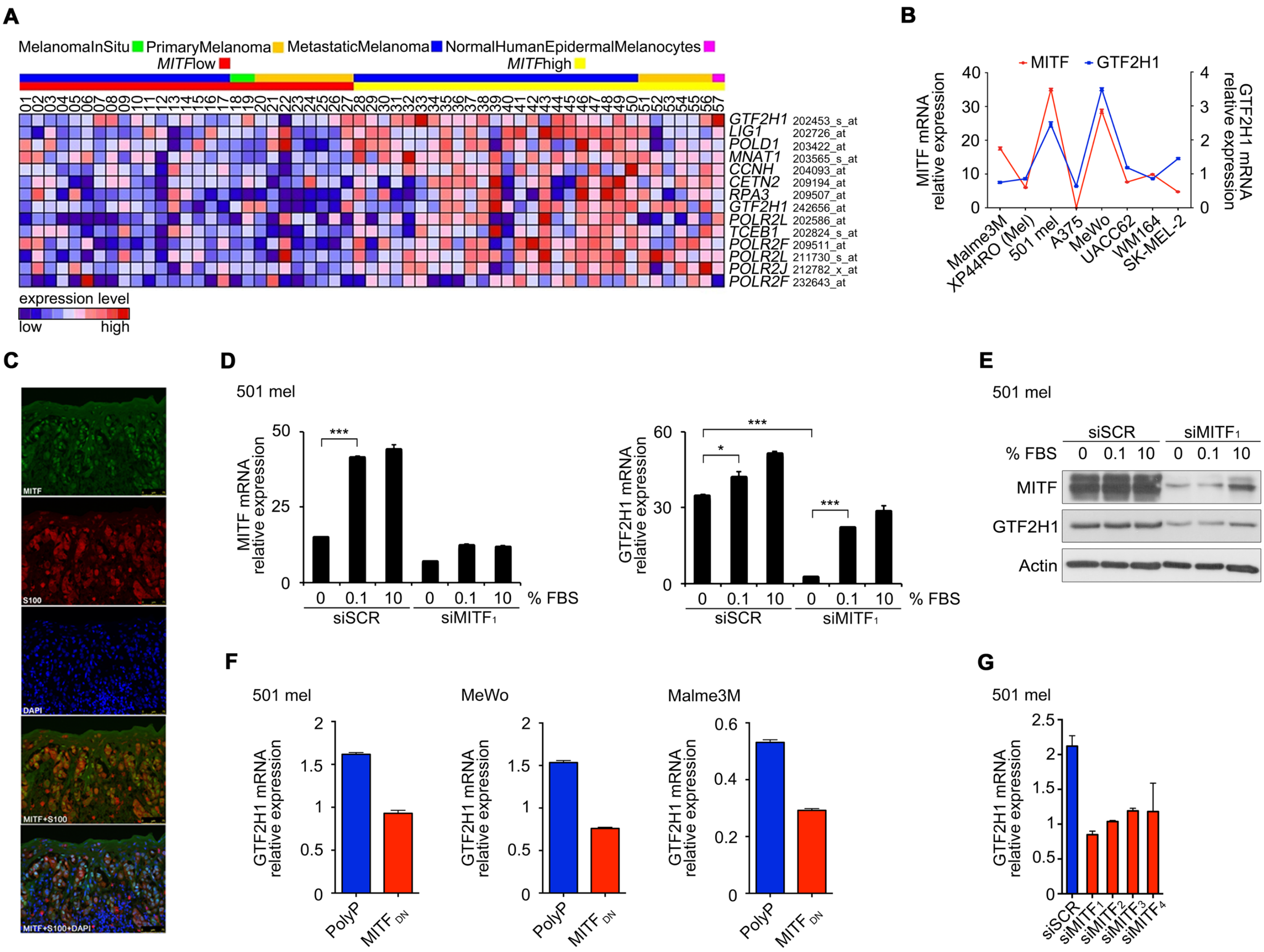

Supplementary Figure 2. MITF-dependent regulation of GTF2H. **a**. Expression heat map of transcriptome-wide comparative marker selection analysis in primary and metastatic melanomas (including melanoma *in situ* and normal melanocytes) based on high versus low MITF expression. Gene symbol and probe set ID are presented. Color bar represents expression level. **b**. MITF and GTF2H1 transcript levels in genetically heterogeneous human melanoma cell lines. Relative expression was measured by qRT-PCR, normalized to GAPDH and given as mean ±SD from triplicates. Y-axis, left: MITF expression; Y-axis, right: GTF2H1 expression. **c**. Immunofluorescence microscopy analysis of cutaneous melanoma tissue sections co-stained for S-100 (red) and MITF (green) to revalidate chromogenic MITF staining in primary cutaneous melanomas (n=136) spotted on TMA (see Fig. 1e). Nuclear counterstaining performed with DAPI (blue). **d**. MITF and GTF2H1 mRNA expression after siSCR or siMITF$_1$ RNA transfection in 501 mel cells at different concentrations of fetal bovine serum (FBS). Graphs represent mean ±SD from triplicates normalized to GAPDH (two-tailed unpaired t-test; *, $p<0.05$; ***, $p<0.001$). **e**. Immunoblot analysis of MITF and GTF2H1 of 501 mel cells treated as in (**d**). Actin used as loading control. **f**. GTF2H1 mRNA expression in three different melanoma cell lines after expression of adenovirus-driven dominant-negative MITF (MITF$_{DN}$) mutant. Relative expression was measured by qRT-PCR, normalized to GAPDH and given as mean ±SD from triplicates. **g**. GTF2H1 mRNA expression after transfection of four different siMITF$_{1-4}$ RNA vs. siSCR in 501 mel cells. Relative expression was measured by qRT-PCR, normalized to GAPDH and given as mean ±SD from triplicates.
