# Supplementary material for: Lineage-specific control of TFIIH by MITF determines transcriptional homeostasis and DNA repair
Source: Oncogene. 2019 Jan 16;38(19):3616–35. doi: 10.1038/s41388-018-0661-x (PMC6756118; doi:10.1038/s41388-018-0661-x)
Supplement: Supplementary file 3 — Supplementary Figure 3 [file 41388_2018_661_MOESM3_ESM.pdf]

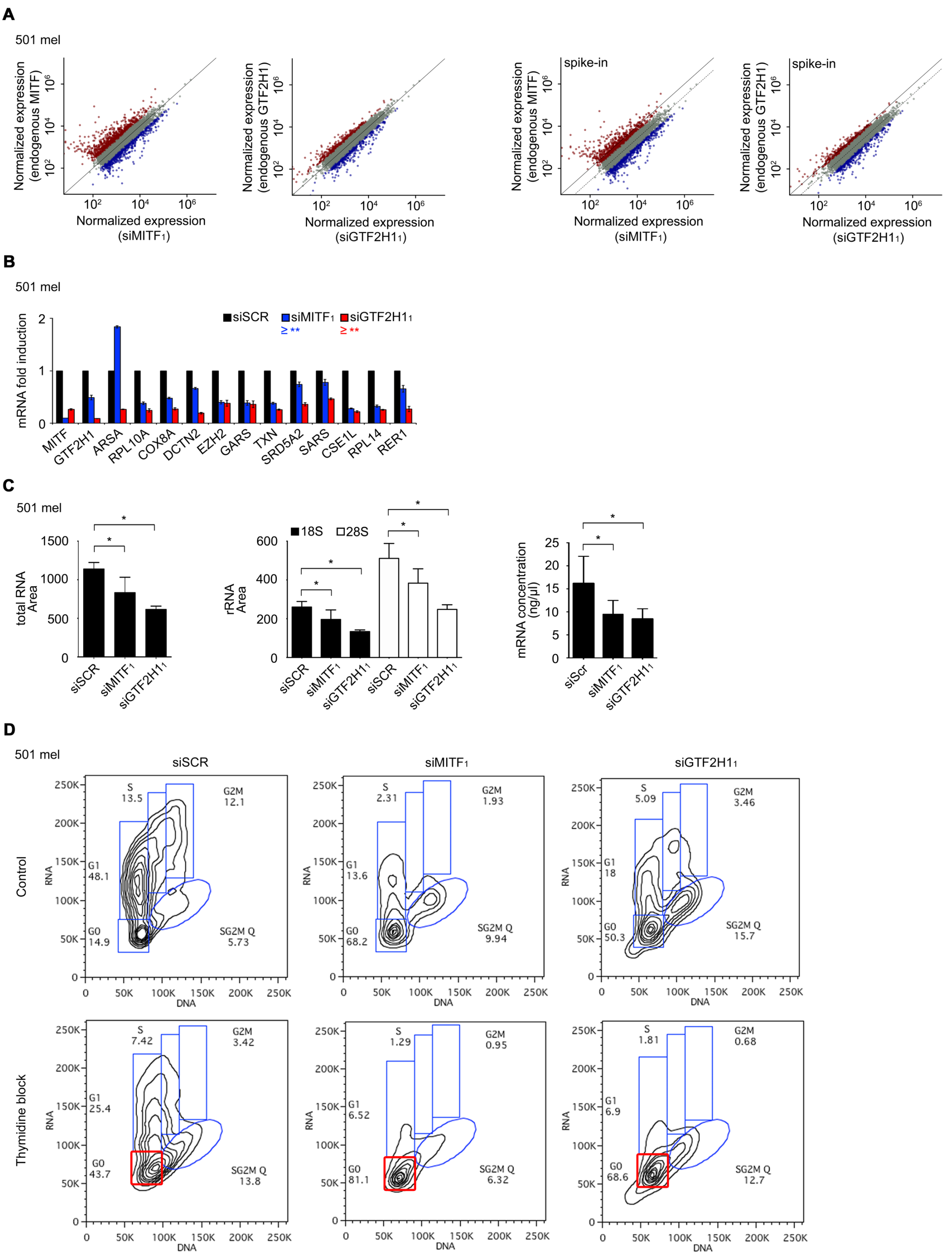

Supplementary Figure 3. MITF-GTF2H1 axis determines global transcriptional output. **a**. RNA-seq analysis of 501 mel cells under siMITF$_1$ or siGTF2H1$_1$ knockdown vs. siSCR (y-axis, endogenous MITF or GTF2H1) normalized to RNA content (left panels) vs. cell equivalents using spike-in RNA (right panels). Expression shift under spike-in conditions indicated by diagonal line. **b**. Expression of randomly selected mRNA transcripts, not qualifying as bona fide MITF target genes, after siRNA transfection of 501 mel cells measured by qRT-PCR. Graph represents mean fold-induction ±SD normalized to GAPDH and cell number from triplicates of two independent experiments. **c**. Quantification of total RNA yield, ribosomal RNA content (18S and 28S) and polyA-hybridized mRNA from siRNA transfected 501 mel cells. Graphs represent mean ±SD normalized to cell number from biological triplicates (two-tailed unpaired t-test; *, $p<0.05$). **d**. FACS based cell cycle analysis of 501 mel cells under siSCR, siMITF$_1$ or siGTF2H1$_1$ transfection and subsequent thymidine block using acridine orange staining to discriminate G0 from G1 cells (G0, red boxes).
