# Supplementary material for: Lineage-specific control of TFIIH by MITF determines transcriptional homeostasis and DNA repair
Source: Oncogene. 2019 Jan 16;38(19):3616–35. doi: 10.1038/s41388-018-0661-x (PMC6756118; doi:10.1038/s41388-018-0661-x)
Supplement: Supplementary file 4 — Supplementary Figure 4 [file 41388_2018_661_MOESM4_ESM.pdf]

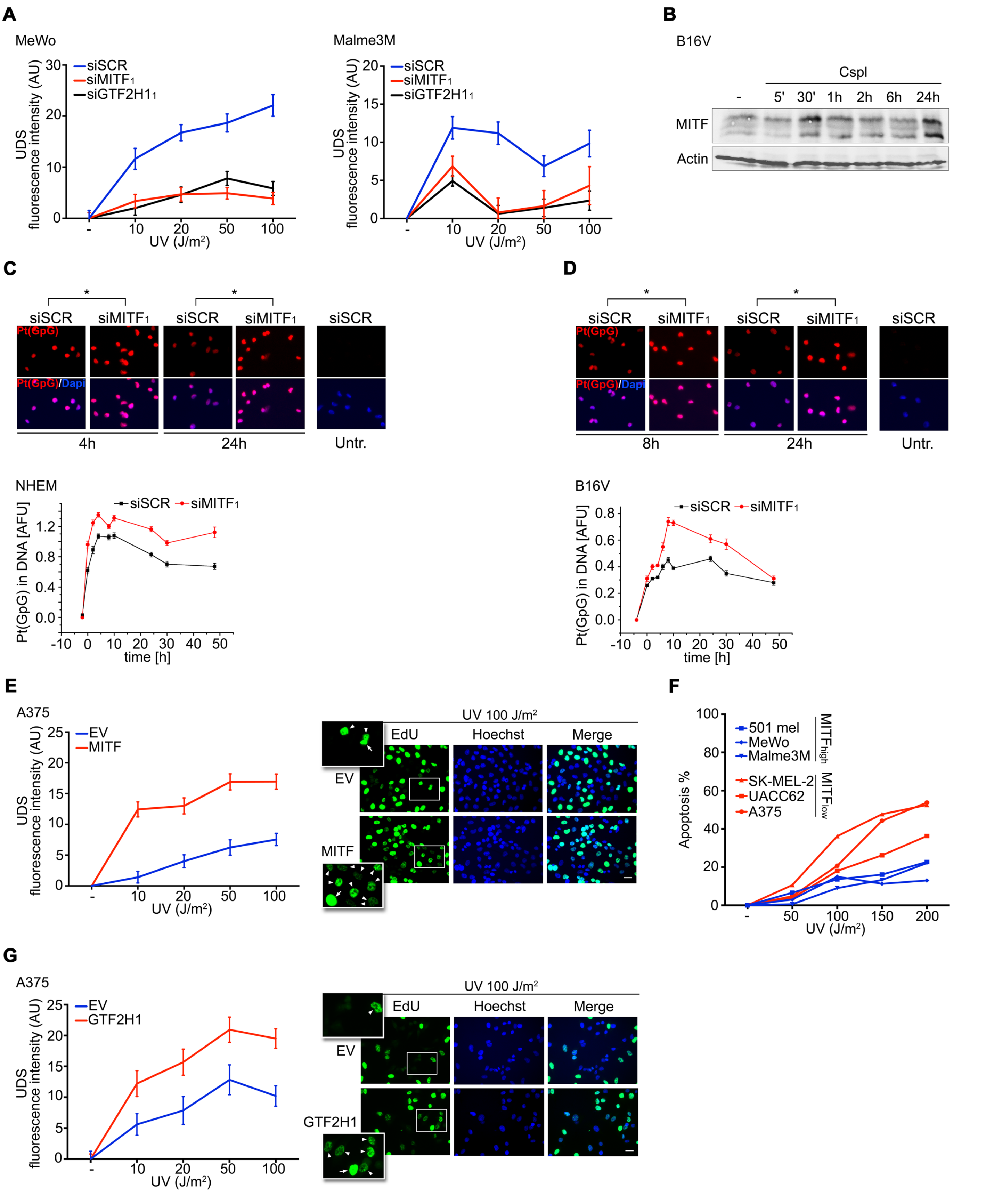

Supplementary Figure 4. Repair of UVR- or cisplatin induced DNA lesions is dependent on MITF. **a**. Unscheduled DNA synthesis (UDS) assay in UV-treated MeWo and Malme3M cell lines after siMITF$_1$ or siGTF2H1$_1$ vs. siSCR RNA transfection. Graphs indicate mean ±SEM of fluorescence intensity in ≥150 non-replicating nuclei for each UV-dosage. **b**. Immunoblot analysis of whole cell protein lysates showing MITF expression in murine B16V melanoma cell line over time upon continuous exposure to cisplatin (6 μg/ml) as indicated. Actin used as loading control. **c**. Immunofluorescence microscopy using an antibody directed against cisplatin-induced Pt-(GpG) intrastrand adducts in nuclear DNA of human primary melanocytes (NHEM) transfected with siMITF$_1$ vs. siSCR. Cells were exposed to cisplatin (20

µg/ml) for 4 hrs and further cultivated in drug-free media for a maximum of 48 hrs. Cell aliquots were analyzed for DNA-adduct levels by *in situ* staining with R-C18 antibody and quantitative ICA analysis. Arbitrary fluorescence units (AFU) values represent means of >100 nuclei ± 95% CI (*). (blue: DAPI [DNA]; red: [Pt-(GpG)] Cy3 antibody). **d**. Immunofluorescence microscopy of transfected B16V murine melanoma cells in analogy to C. **e**. UV-induced UDS assay in MITF-negative A375 cells after retroviral expression of wildtype MITF or empty vector (EV). Graph indicates mean ±SEM of fluorescence intensity in ≥200 non-replicating nuclei for each UV-dosage. Right panel shows representative micrographs of irradiated cells at 50 J/m^2. (Insets: Arrowhead, UDS; arrows, replicating cells; scale: 25 µm). **f**. Apoptosis analysis by FACS annexinV/propidium iodide staining of melanoma cells with low/negative (Sk-MEL-2, UACC62 and A375) or high (501 mel, MeWo and Malme3M) abundance of MITF after UVB-irradiation at the indicated doses. **g**. UDS assay in MITF-negative A375 cells after retroviral expression of GTF2H1 or empty vector (EV) in analogy to (**e**).
