# Supplementary material for: Lineage-specific control of TFIIH by MITF determines transcriptional homeostasis and DNA repair
Source: Oncogene. 2019 Jan 16;38(19):3616–35. doi: 10.1038/s41388-018-0661-x (PMC6756118; doi:10.1038/s41388-018-0661-x)
Supplement: Supplementary file 5 — Supplementary Figure 5 [file 41388_2018_661_MOESM5_ESM.pdf]

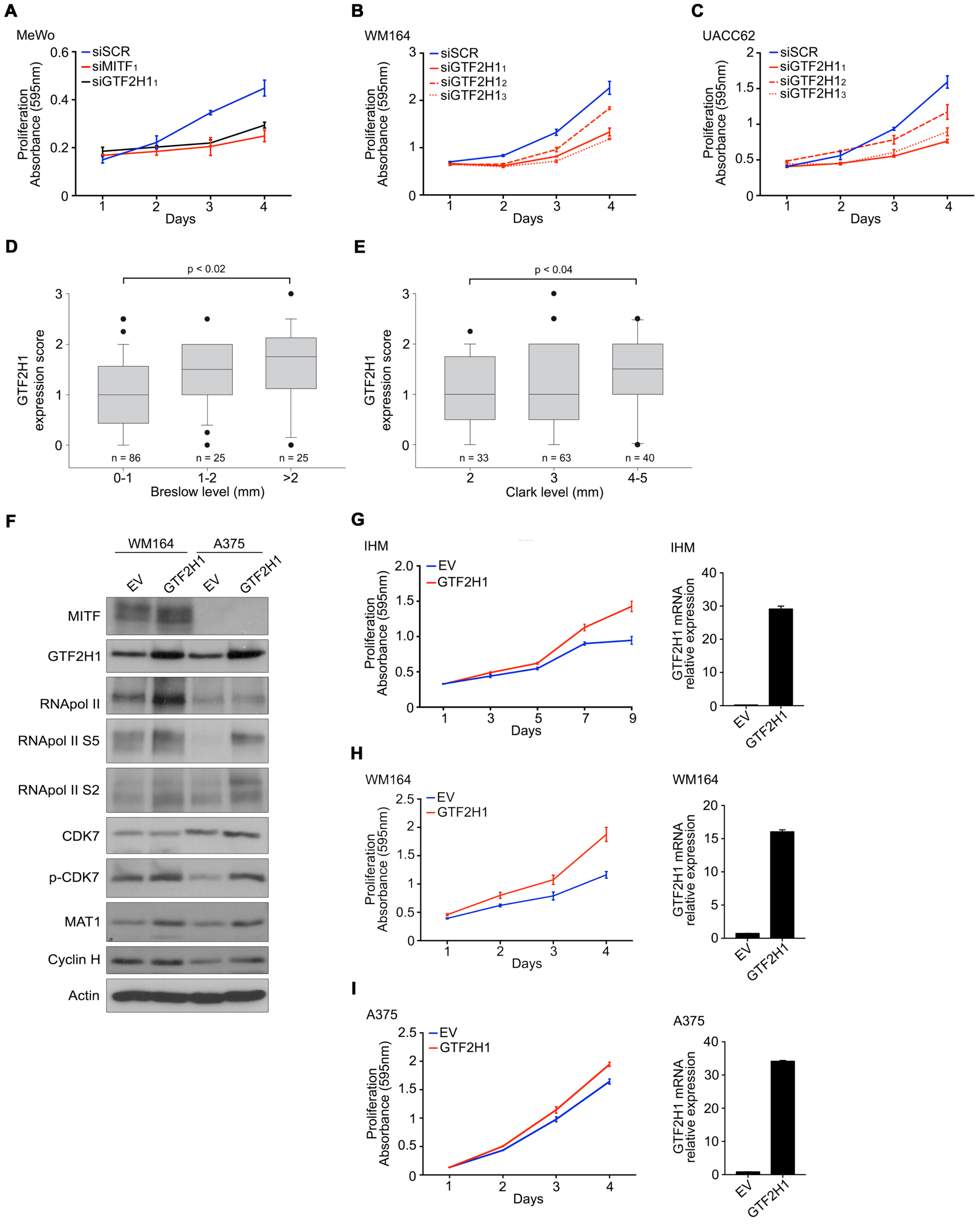

Supplementary Figure 5. GTF2H1 determines transcriptional activity and affects growth of the melanocytic lineage including melanoma. **a**. Proliferation of MeWo cells after $siMITF_1$ or $siGTF2H1_1$ transfection vs. siSCR control using crystal violet (CV) dye assay. Graph represents mean ±SD of CV absorbance from technical triplicates. **b**. Proliferation of WM164 cells after siRNA transfection with

three different siGTF2H1$_{1\text{-}3}$ vs. siSCR. Graph represents mean ±SD of CV absorbance from technical triplicates. **c**. Proliferation of UACC62 cells in analogy to (**b**). **d**. Box plot shows the relationship between GTF2H1 expression score and tumor thickness/diameter (Breslow level) (score: 0, negative; 0≤1.5, low; >1.5≤2.5, intermediate; >2.5 high expression). Horizontal line in box plots represents the mean. n, number of samples grouped according to Breslow level. **e**. Box plot shows the relationship between semi-quantitative GTF2H1 expression and Clark level (representing depth of invasion) of primary cutaneous melanomas. **f**. Phospho-immunoblot analysis of POLR2 and TFIIH components in WM164 and A375 cellular lysates under retrovirus-driven GTF2H1 expression or empty vector (EV). Actin used as loading control. **g**. Proliferation of immortalized human melanocytes (IHM) cells upon forced retroviral expression of GTF2H1 vs. empty vector (EV). Graph represents mean ±SD of CV absorbance from technical triplicates. Right panel shows GTF2H1 transcript levels in IHM under retrovirus-driven GTF2H1 expression compared to empty vector control (EV). Relative expression was measured by qRT-PCR, normalized to GAPDH and given as mean ±SD from triplicates. **h**. Proliferation and GTF2H1 transcript levels of WM164 cells in analogy to (**g**). **i**. Proliferation and GTF2H1 transcript levels of A375 cells in analogy to (**h**).
