# Supplementary material for: Lineage-specific control of TFIIH by MITF determines transcriptional homeostasis and DNA repair
Source: Oncogene. 2019 Jan 16;38(19):3616–35. doi: 10.1038/s41388-018-0661-x (PMC6756118; doi:10.1038/s41388-018-0661-x)
Supplement: Supplementary file 6 — Supplementary Figure 6 [file 41388_2018_661_MOESM6_ESM.pdf]

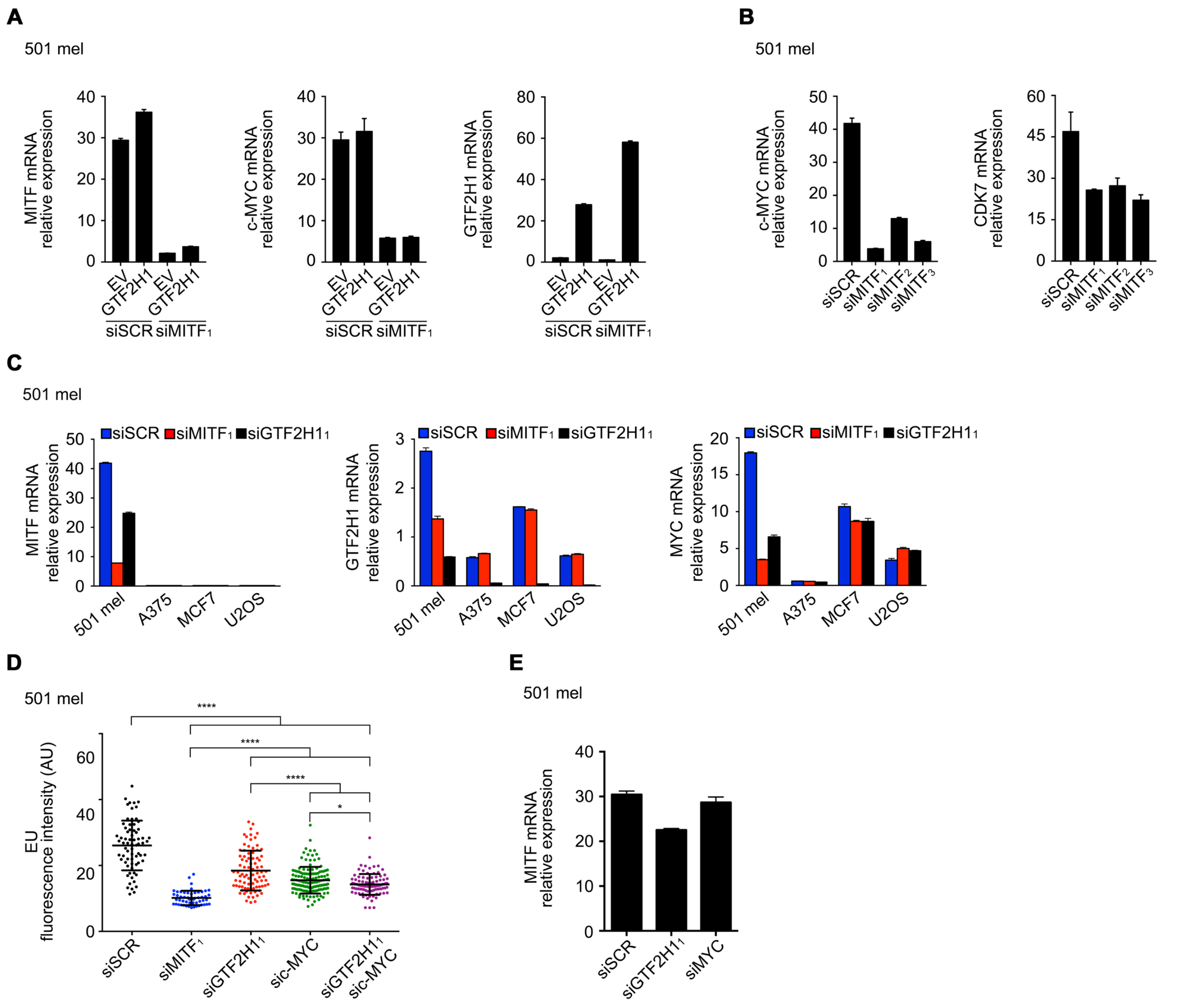

Supplementary Figure 6. Dominant role of MITF in the regulation of transcriptional homeostasis. **a**. MITF, c-MYC and GTF2H1 transcript levels in 501 mel cells under retrovirus-driven GTF2H1 or empty vector (EV) expression and subsequent siSCR or siMITF$_1$ RNA transfection. Relative expression was measured by qRT-PCR, normalized to GAPDH and given as mean ±SD from technical triplicates. **b.** Expression of MYC and CDK7 transcripts under RNAi using independent siMITF$_{1-3}$ compared to siSCR control measured as in (**a**). **c**. Expression of MITF, GTF2H1, and MYC transcripts in MITF-dependent 501 mel cells, MITF-negative A375 cells and non-melanocytic cell lines (MCF7, U2OS) upon MITF- or GTF2H1-directed siRNA transfection. Relative expression was measured by qRT-PCR, normalized to GAPDH and given as mean ±SD from technical triplicates. **d**. Analysis of transcriptional activity by 5-ethynyl-uridine (EU) incorporation in 501 mel cells after siSCR, siMITF$_1$, siGTF2H1$_1$, siMYC or siMYC combined with siGTF2H1$_1$ transfection. Scatter dot plot represents fluorescence intensity of discrete nuclei. Error bars indicate mean ±SEM of fluorescence intensity of ≥100 nuclei (two-tailed unpaired t-test; *, $p<0.05$; ****, $p<0.0001$). **e**. MITF transcript levels in 501 mel cells after siSCR, siGTF2H1$_1$ or siMYC transfection. Relative expression was measured by qRT-PCR, normalized to GAPDH and given as mean ±SD from technical triplicates.
