# Supplementary material for: Lineage-specific control of TFIIH by MITF determines transcriptional homeostasis and DNA repair
Source: Oncogene. 2019 Jan 16;38(19):3616–35. doi: 10.1038/s41388-018-0661-x (PMC6756118; doi:10.1038/s41388-018-0661-x)
Supplement: Supplementary file 7 — Supplementary Figure 7 [file 41388_2018_661_MOESM7_ESM.pdf]

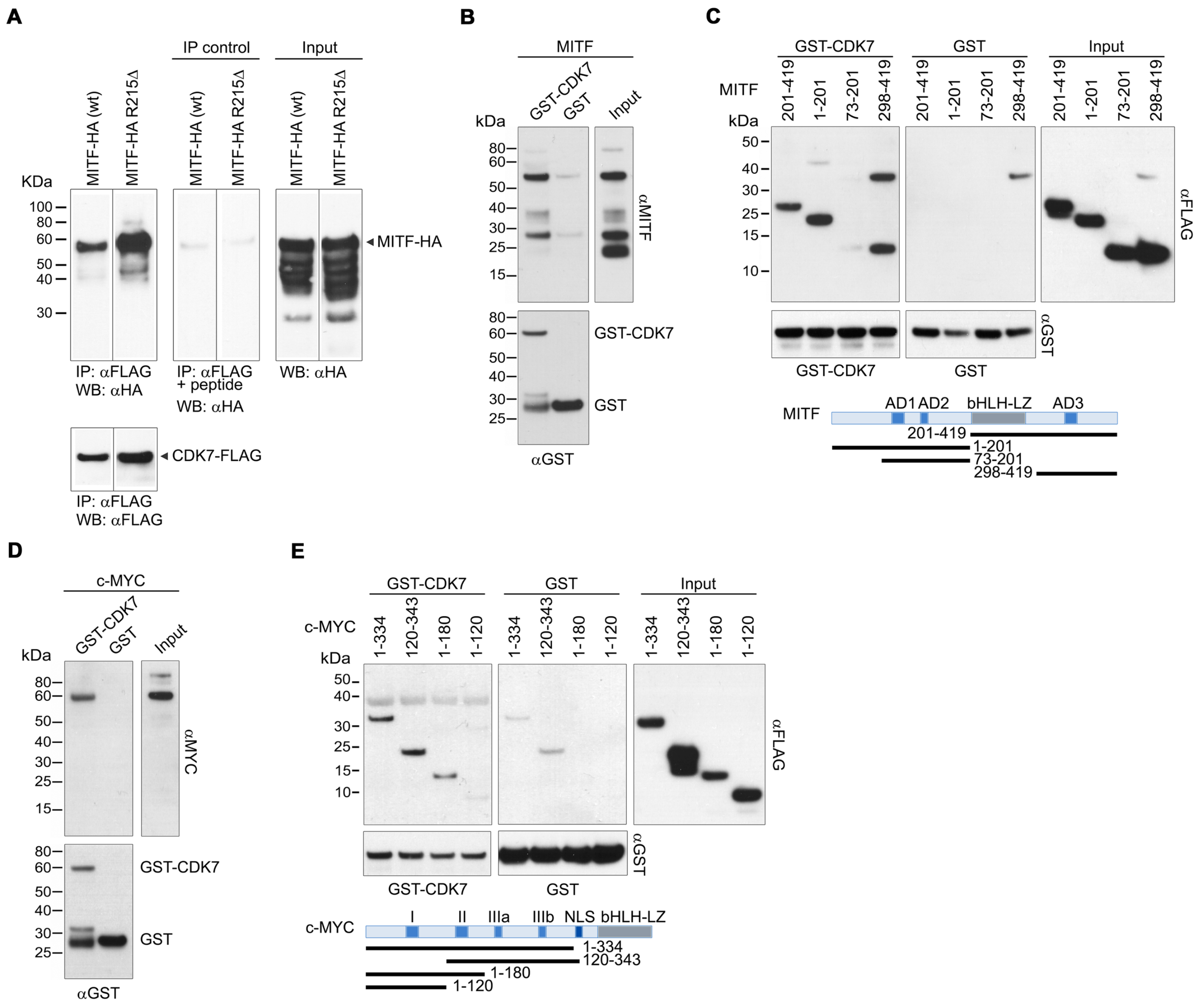

Supplementary Figure 7. MITF and c-MYC bind CDK7. **a**. *In vivo* binding assay of CDK7-FLAG and MITF-HA wildtype or DNA-binding-domain (DBD)-mutated MITF-HA R215Δ upon transfection of HEK293T cells. CDK7-FLAG detection used as loading control. Immunoprecipitation control was performed using anti-FLAG antibody in the presence of FLAG-peptide. Input was analyzed by immunoblot using anti-HA antibody. **b**. *In vitro* binding assay of MITF with GST-CDK7 upon prokaryotic expression. MITF was detected using anti-MITF C5 antibody. Abundance of GST and GST-CDK7 was determined by anti-GST antibody. **c**. Analysis of protein-protein-interaction between CDK7 and MITF by *in vitro* binding assay of GST-CDK7 with different FLAG-tagged MITF deletion mutants. Detection of MITF-mutants binding to GST-CDK7 performed by anti-FLAG antibody. GST used as loading control. Schema represents functional domains of MITF and aligned recombinant deletion mutants. AD1-3, transactivation domains were marked in light blue; bHLH-LZ, basic helix-loop-helix-leucine zipper, was marked in dark gray. **d**. Analysis of protein-protein interaction between CDK7 and MYC by *in vitro* binding assay of wildtype c-MYC with GST-CDK7. Experimental setup was analogous to (**b**). **e**. *In vitro* binding assay of GST-CDK7 with different FLAG-tagged c-MYC deletion mutants in analogy to (**c**). GST used as loading control. Schema represents functional domains of c-MYC and aligned recombinant deletion mutants. I-IIIa, b MYC boxes; NLS, nuclear localization signal.
