# Supplementary material for: Lineage-specific control of TFIIH by MITF determines transcriptional homeostasis and DNA repair
Source: Oncogene. 2019 Jan 16;38(19):3616–35. doi: 10.1038/s41388-018-0661-x (PMC6756118; doi:10.1038/s41388-018-0661-x)
Supplement: Supplementary file 8 — Supplementary Figure 8 [file 41388_2018_661_MOESM8_ESM.pdf]

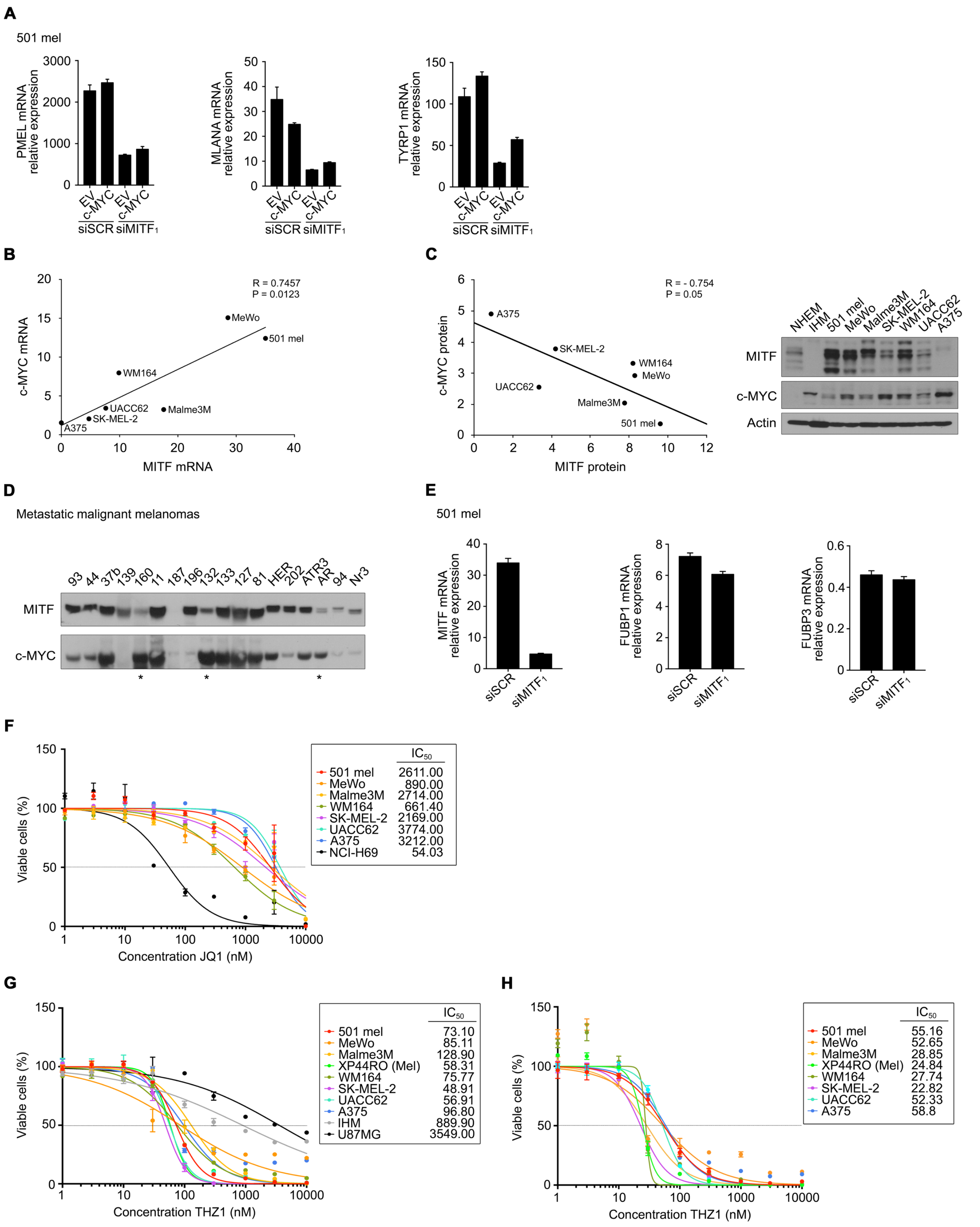

Supplementary Figure 8. Transcription dependency of melanoma on MITF or MYC sensitizes toward TFIIH-CAK inhibition. **a**. Expression analysis of PMEL, MLANA and TYRP1 transcripts in 501 mel cells under retrovirus-driven c-MYC expression compared to empty vector (EV) and subsequent siSCR or $siMITF_1$ RNA transfection. Relative expression was measured by qRT-PCR, normalized to GAPDH

and given as mean ±SD from technical triplicates. **b**. Regression analysis of MITF and c-MYC transcript levels in genetically heterogeneous human melanoma cell lines. Relative expression was measured by qRT-PCR and normalized to GAPDH. **c**. Regression analysis of MITF and c-MYC protein levels in analogy to (**b**). Right panel: Immunoblot analysis of whole cell lysates showing MITF and c-MYC protein in primary neonatal human epidermal melanocytes (NHEM), immortalized human melanocytes (IHM), and various human melanoma cell lines analyzed analogous to (**b**). Actin used as loading control. **d**. Immunoblot analysis of whole cell lysates from n=18 human metastatic malignant melanomas exhibiting MITF and c-MYC protein expression. The asterisk (*) indicates tumors with low MITF/c-MYC expression ratio. Sample 187 was not quantifiable. **e**. Expression analysis of MITF and FUBP isoforms 1 and 3, which do not contain E box sequences in regulatory regions in contrast to FUBP2 (Fig 6G), upon MITF-directed RNAi using $siMITF_1$ in 501 mel cells. Relative expression was measured by qRT-PCR, normalized to GAPDH and given as mean ±SD from technical triplicates. **f**. WST-1 based cell cytotoxicity assay under treatment with BET bromodomain inhibitor JQ1 for 48 hrs of a diverse set of melanoma cell lines compared to NCI-H69 lung carcinoma cell line as positive JQ1 sensitive control. **g**. WST-1-based cell cytotoxicity assay of a panel of genetically heterogeneous melanoma lines after treatment with covalent CDK7 inhibitor THZ1 for 48 hrs. Immortalized human melanocytes (IHM) and U87MG glioblastoma cells as THZ1-resistant controls. **h**.WST-1 based cell cytotoxicity assay of a panel of genetically heterogeneous melanoma lines after treatment with covalent CDK7 inhibitor THZ1 for 72 hrs. (**f**), (**g**) and (**h**); Graphs represent percentage cell viability relative to DMSO control. Data represent mean ±SD from technical triplicates.
