# Supplementary material for: Lineage-specific control of TFIIH by MITF determines transcriptional homeostasis and DNA repair
Source: Oncogene. 2019 Jan 16;38(19):3616–35. doi: 10.1038/s41388-018-0661-x (PMC6756118; doi:10.1038/s41388-018-0661-x)
Supplement: Supplementary file 9 — Supplementary Figure 9 [file 41388_2018_661_MOESM9_ESM.pdf]

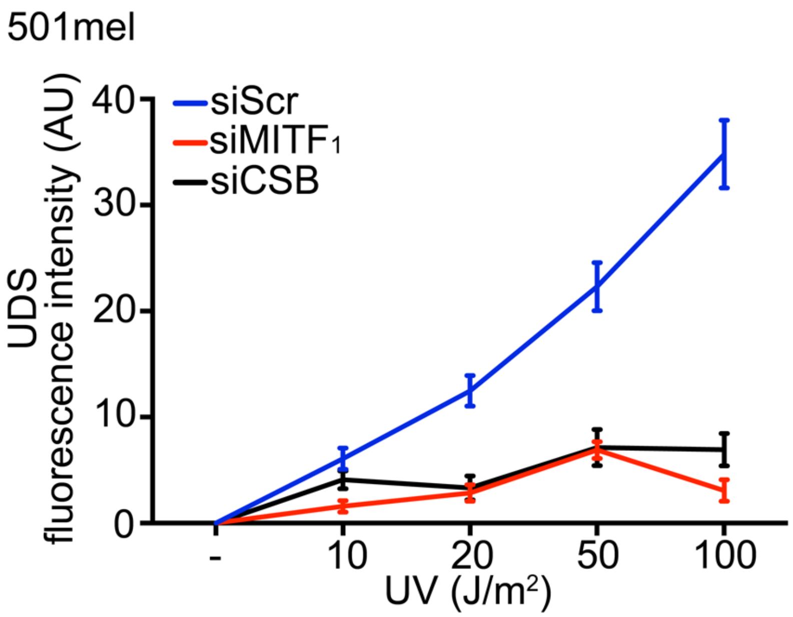

Supplementary Figure 9. Inhibition of TCR-specific CSB recapitulates NER deficiency by MITF depletion. UV-induced UDS assay in 501 mel cells after siMITF$_1$ or siCSB (TCR-specific Cockayne syndrome protein B helicase) vs. siSCR RNA transfection. Graph indicates mean ±SEM of fluorescence intensity in ≥200 non-replicating nuclei for each UV-dosage.
