# Supplementary material for: Lineage-specific control of TFIIH by MITF determines transcriptional homeostasis and DNA repair
Source: Oncogene. 2019 Jan 16;38(19):3616–35. doi: 10.1038/s41388-018-0661-x (PMC6756118; doi:10.1038/s41388-018-0661-x)
Supplement: Supplementary file 10 — Supplementary Table 1 [file 41388_2018_661_MOESM10_ESM.pdf]

Supplementary Table 1. Genetic profile of melanoma cell lines.

Melanoma cell line	BRAF	PTEN	N-ras	c-KIT	CDK4	CDKN2A (p16)	p53	MITF Copies
**A375**	V600E	WT	WT				WT	
**Mewo**	WT		WT				WT/ Mut #	
**Malme 3M**	V600E					HD		6
**XP44RO (Mel) ***								
**SK-MEL-2**	WT		G12D			WT	Mut	2
**501 mel**	V600E							
**WM164**	V600E	WT	WT	WT	WT			
**UACC62**	V600E	Mut				HD		

Hem Del = Hemizygous deletion
HD = Homozygous deletion
* = XPC deficient
# = controversial data
