# Supplementary material for: Lineage-specific control of TFIIH by MITF determines transcriptional homeostasis and DNA repair
Source: Oncogene. 2019 Jan 16;38(19):3616–35. doi: 10.1038/s41388-018-0661-x (PMC6756118; doi:10.1038/s41388-018-0661-x)
Supplement: Supplementary file 11 — Supplementary Table 1 [file 41388_2018_661_MOESM11_ESM.pdf]

Supplementary Table 2. Clinicopathological characteristics regarding primary cutaneous melanomas on TMA.

	n	%
**Number of patients**	140	100
Female	65	46.4
Male	72	51.4
Unknown	3	2.2
**Age**		
Age at Diagnosis (mean)	54 y	
Age at Diagnosis (range)	20-94 y	
**Tumor thickness (Breslow)**		
<=1	92	65.7
1.01-2	22	15.7
2.01-4	12	8.6
>4	10	7.1
unknown	4	2.9
**Clark level**		
I	0	0
II	33	23.5
III	63	45.0
VI	34	24.3
V	6	4.3
unknown	4	2.9
**Growth pattern**		
SSM	105	75.0
NMM	18	12.9
LMM	9	6.4
ALM	2	1.4
NOS	6	4.3
**Ulceration**		
No	131	93.6
Yes	6	4.3
unknown	3	2.1

Primary cutaneous melanoma (MM) material of patients above was collected between 1994 and 2006 and consists of consecutive (non-selected) MM samples from 140 patients of the Department of Dermatology and Venerology, University Medical Center Hamburg-Eppendorf, Germany. It contained two tissue cores per melanoma specimen. For patients with multiple subsequent neoplasms, only initial and single primary MMs were included. Abbreviations: SSM, superficial spreading melanoma; NMM, nodular MM; LMM, lentigo maligna melanoma; ALM, acral lentiginous melanoma; NOS, malignant melanoma of skin.
